# A perfect storm: the immunological and pathophysiological landscape of pediatric post-COVID-19 condition

**DOI:** 10.3389/fimmu.2026.1794596

**Published:** 2026-04-10

**Authors:** Coen R. Lap, Marlies van Houten, Debby Bogaert, Giske Biesbroek

**Affiliations:** 1 Department of Pediatrics, Spaarne Gasthuis, Haarlem, Netherlands; 2 Department of Pediatric Pulmonology, Emma Kinderziekenhuis Amsterdam University Medical Center (UMC), Amsterdam, Netherlands; 3 Universiteit Utrecht Departement Infectieziekten en Immunologie, Utrecht, Netherlands; 4 Universitair Medisch Centrum Utrecht - Locatie Wilhelmina Kinderziekenhuis, Utrecht, Netherlands; 5 The University of Edinburgh Centre for Inflammation Research, Edinburgh, United Kingdom; 6 Rijksinstituut voor Volksgezondheid en Milieu, Bilthoven, Netherlands

**Keywords:** immune dysregulation, latent viral reactivation, metabolic dysfunction, microbiome dysbiosis, neuroinflammation, pediatric post COVID condition, vascular inflammation, viral persistence

## Abstract

Pediatric Post-COVID Condition (PPCC) represents a significant and complex long-term sequela of SARS-CoV-2 infection, affecting a subset of children and adolescents even after mild acute disease. While acute COVID-19 is generally milder in children due to a more robust innate immune response, the mechanisms driving the persistence of symptoms in PPCC remain incompletely understood and likely multifactorial. This narrative review synthesizes current epidemiological data and explores the “perfect storm” of immunological and pathophysiological alterations underpinning the condition. We examine critical hypotheses including a dysregulated immune response characterized by altered T-cell subsets, monocyte activation, and autoantibody production. We discuss the potential role of persistent SARS-CoV-2 viral reservoirs in “sanctuary sites” like the gastrointestinal tract and the reactivation of latent viruses such as Epstein-Barr virus (EBV). Furthermore, the review details downstream pathogenic pathways, including vascular endothelial inflammation (thrombo-inflammation), neuroinflammation, and metabolic dysfunctions affecting the mitochondria and tryptophan-kynurenine pathway. Finally, we address the role of microbiome dysbiosis in perpetuating systemic inflammation and the gut-lung axis dysfunction. Given the heterogeneity of clinical presentations, we conclude that PPCC is likely a syndrome of overlapping biological phenotypes. Future research must prioritize identifying these specific biological endotypes to develop targeted diagnostic and therapeutic strategies for the pediatric population.

## 1 Introduction: pediatric post-COVID condition

While acute COVID-19 infection tends to be milder in children than in adults, the long-term effects of SARS-CoV-2 infection, termed long COVID or post-COVID condition (PCC), can still affect children (1–5). Children who have experienced a more severe acute COVID-19 disease course have a higher prevalence of persistent symptoms (6–8). Reported prevalence rates of PPCC vary considerably, ranging from lower estimates of 1.5% to a pooled prevalence of 23.36% reported in a meta-analysis (6–13). A notable UK study, conducted by the CLoCk consortium, compared the symptom profiles of SARS-Cov2 positive children (aged 11-17) to a test-negative control group. This study found that several symptoms, including abdominal pain, diarrhea, chest pain, and confusion, were more common in SARS-CoV-2-positive children (11–17 years) during the acute phase. Although the frequency of these symptoms generally declined at three and six months post-infection (14, 15). A subset of children continued to experience persistent complaints. The impact of PPCC in daily life underscores the need for comprehensive research into diagnostic criteria, pathobiology or underlying pathophysiology, and targeted treatment strategies.

This narrative review summarizes current studies focused on PPCC by their epidemiology and symptoms. Multiple potential pathogenic pathways have been described in PCC, including a dysregulated immune response, a persistent viral reservoir, reactivation of latent viruses, vascular (endothelial) inflammation, neuronal inflammation and dysfunction, metabolic/mitochondrial dysfunction, and microbiome dysbiosis. Given the scarcity of pediatric specific data, we incorporate relevant findings from adult PCC studies to provide context and support hypothesis generation. Where pediatric data are lacking, adult models may offer provisional insights into underlying biological processes. In the final section of this review, we propose a conceptual model to illustrate the multifactorial nature of post-COVID complexity in children. We also identify key gaps in current research and outline priorities for future studies aimed at improving diagnosis, management, and long-term outcomes.

### 1.1 Definition and prevalence of pediatric post-COVID condition

Pediatric post-COVID condition (PPCC) refers to the persistence of symptoms for at least two months following a SARS-CoV-2 infection in children and adolescents. While “long COVID” is a broader term for such persistent symptoms in any age group (16, 17), PPCC is defined using the World Health Organization (WHO) criteria for post-COVID condition (PCC). These criteria specify the presence of symptoms for at least two months that initially occurred within three months of acute COVID-19 (18, 19). Such symptoms typically impact daily functioning, can be new or a continuation of the initial illness, and may fluctuate or relapse over time (18).

Children can develop PPCC even after a mild or asymptomatic COVID-19 infection. This condition can also occur following reinfection, potentially triggering new post-COVID symptoms or exacerbating existing ones (11, 20–23). Studies demonstrate that prevalence decreases with time; 20% of children experience persistent symptoms at six months, dropping to 4.4% to 11% at 12 months (8, 9, 24). However, there are significant differences in the reported severity of PPCC cases.

Post-COVID is a multi-organ, multisystem condition, causing a wide range of symptoms (13, 20). A meta-analysis of post-COVID symptoms in children and young people (CYP) reported pooled prevalences for fatigue (47%), headache (35%), and dyspnea (43%) as the most prevalent symptoms (25). Other research indicates that the prevalence of fatigue in CYP with post-COVID may vary significantly, with for example the PoCoCoChi study reporting lower prevalence rates of fatigue (around 10%), possibly correlating with increased age (8). In a meta-analysis, cognitive difficulties, myalgia, and abdominal pain were reported by 26%, 25%, and 25% of CYP, respectively, while anosmia/altered sense of smell and fever were both reported at 18% (25), and cough (17%) and diarrhea (15%) were reported least frequently (25). Another study found that a high percentage of children with post-COVID met the diagnostic criteria for postural orthostatic tachycardia syndrome (POTS) (26), a common cardiovascular autonomic dysfunction, also commonly reported in adult PCC (27, 28).

### 1.2 Risk factors for PPCC

While a substantial proportion of individuals with post-COVID have no prior health conditions – approximately two-thirds of children and one-third of adults (3, 29) – several factors can increase the risk of developing Pediatric Post-COVID Condition (PPCC) (**Box 1**). These include acute infection severity and number of symptoms and organ systems affected during the acute infection, as well as age, sex, weight status, pre-existing medical conditions, and SARS-CoV-2 variant.

Box 1 **Risk factors for PPCC.**

Severity of initial COVID-19 infection: A more severe acute illness in children is linked to a higher likelihood of developing long-COVID symptoms (10–13, 29). This includes children and adolescents who were hospitalized for acute COVID-19 being at greater risk of persistent symptoms (8, 30, 31). The severity of the initial infection is also a recognized risk factor in adults (3, 24, 32–34).

Increasing age: Within pediatric populations, increasing age is a risk factor for post-COVID. This is distinct from, though parallel to, increasing age being a strong predictor in adults across their broader age range (24, 32, 35). School-age children (6–11 years) are more likely to present with neurocognitive issues, pain, and gastrointestinal symptoms. In contrast, adolescents (12–17 years) more commonly experience changes in smell or taste, pain, and fatigue/malaise (36).

Number of symptoms during acute infection: Experiencing a greater number of symptoms during the initial phase of COVID-19 is associated with a higher risk of PPCC in children (10, 11, 13, 29, 37, 38).

Female sex: Similar to findings in adults, female sex has been identified as an associated risk factor for PPCC (10–13, 29).

Weight status: Being overweight or obese is an additional risk factor for developing PPCC, a finding also mirrored in adult populations (10–13, 29).

Pre-existing medical conditions: The presence of pre-existing health conditions, such as asthma, allergies, epilepsy, ADHD, and atopic dermatitis, can elevate the risk of PPCC (37). Any pre-existing medical conditions, more generally, can also influence risk (10, 11, 13, 29).

SARS-CoV-2 variant: The specific viral variant causing the initial infection may influence risk (10, 11, 13, 29).

Number of organ systems affected: The involvement of multiple organ systems during the acute illness can also increase the risk of PPCC (10, 11, 13, 29).

Socioeconomic and environmental factors may contribute to the development of PPCC development, though underlying mechanisms remain unclear (3, 24, 30, 31). Some studies suggest that belonging to certain ethnic minority groups is associated with a higher risk of PPCC; however, it is crucial to consider potential confounding factors, such as socioeconomic disparities, and access to healthcare, which may contribute to this association (3, 24, 30, 31). While SARS-CoV-2 vaccination has been shown to reduce long-term complications in adults (32), its specific effect on PPCC risk is still under investigation. Ongoing research continues to explore the broader influence of biopsychosocial and environmental determinants in post-COVID outcomes (11).

### 1.3 Current hypotheses in pediatric post-COVID condition

**Figure 1:**
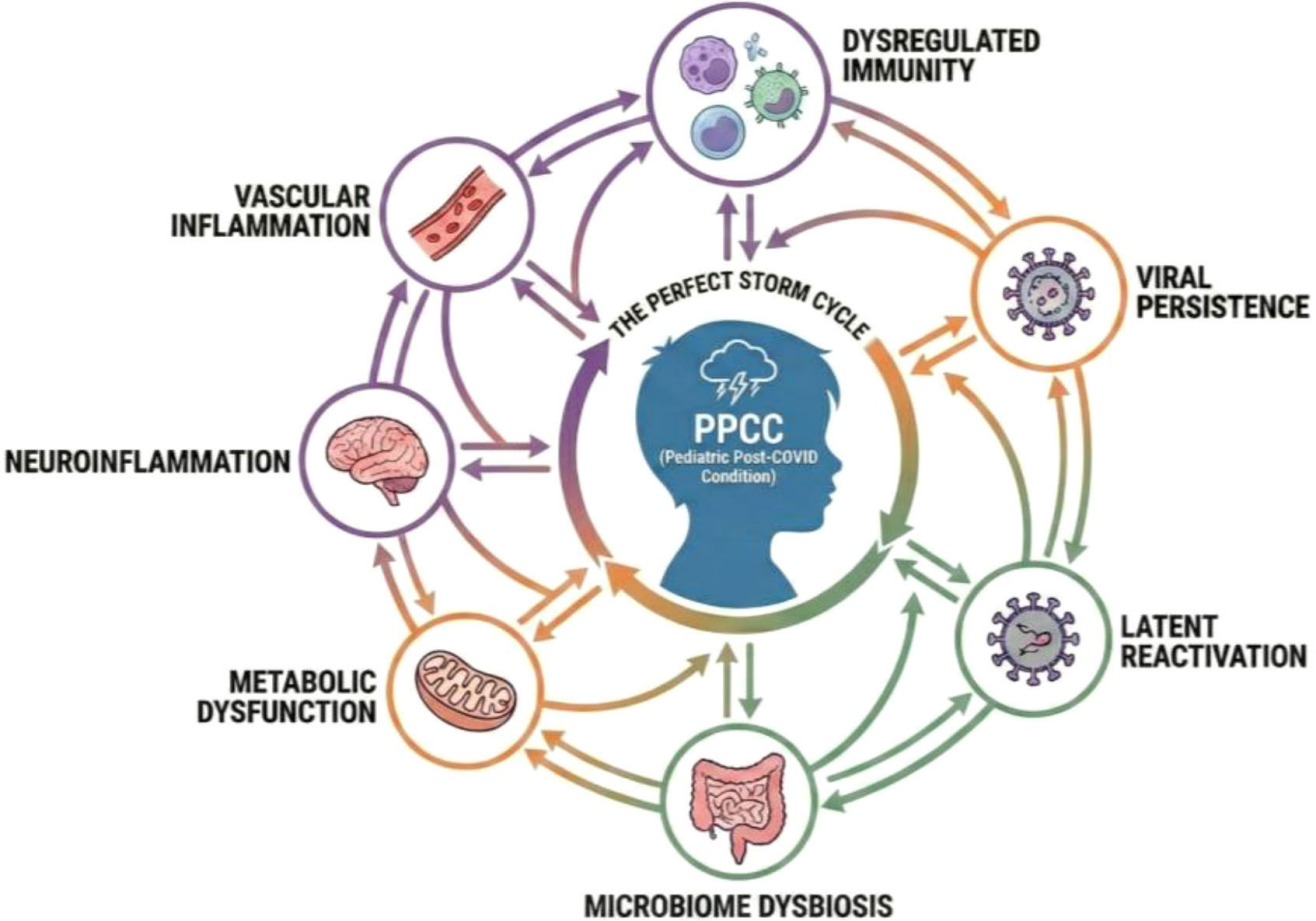
The perfect storm of PPCC.

Although research into the etiology of PPCC is ongoing, current evidence suggests a likely multifactorial origin, similar to that observed in adults (3) (**Figure 1**). A central hypothesis is dysregulation in the immune response, with a potential role for the persistence of SARS-CoV-2 viral reservoirs and triggering of (auto)immune reactions (20, 30, 33–35). Reactivation of latent viruses by COVID-19 infection might be another contributing factor that is currently under investigation (20, 30, 33, 34). Direct tissue damage and dysfunction caused by the initial infection could also contribute to long-term sequelae; however, this mechanism is considered less relevant in children and is therefore not discussed further in this review (20, 30, 33, 35). Mitochondrial dysfunction, vascular (endothelial) inflammation (including microclots and aberrant platelet activation), and neuronal inflammation have also been investigated (20, 33–37). Finally, microbiome dysbiosis has been implicated (20, 33–35). Understanding how these diverse factors interact and contribute to the development and persistence of symptoms in PPCC is crucial for the development of effective treatments.

## 2 Mechanisms of PPCC

### 2.1 Dysregulated immune response

Dysregulation of the immune response has been implicated as an important mechanism in post-COVID condition in general (38, 39). Several components of the immune system seem to be involved, including the emergence of auto-antibodies, and downstream might enable the persistence of SARS-CoV-2 viral reservoirs.

#### 2.1.1 Innate immune system

During the acute phase of a SARS-CoV-2 infection, the innate immune system plays a crucial role in the initial detection of and response to the virus. Upon entering the human body, SARS-CoV-2 is detected by a diverse array of pattern recognition receptors (PRRs), including Toll-like receptors (TLRs) and RIG-I-like receptors (RLRs) (40).

In severe COVID-19, multiple mechanisms within the antiviral response are dysregulated, leading to uncontrolled viral replication, excessive inflammatory responses, and tissue damage (40). Age-related differences in the innate immune response have been proposed as a factor contributing to the generally milder disease course observed in children compared to adults (1, 41–43). For example, Brodin and colleagues have highlighted that children, especially younger children, often exhibit a more robust *local* innate immune response in the upper airways, characterized by higher baseline expression of PRRs and a more rapid induction of interferon-stimulated genes (ISGs) upon viral exposure (14, 44, 45). This enhanced mucosal immunity may contribute to reduced viral replication and a lower likelihood of severe lower respiratory tract involvement in children (14, 44, 45). While children generally mount a strong initial innate response, the overall inflammatory response may be less systemic compared to adults, potentially mitigating the risk of cytokine storm and severe systemic inflammation (14, 41–43).

Studies have reported activated innate immune cells in individuals with post-COVID conditions, underscoring the potential role of the innate immune system in the pathophysiology of these conditions (46). Specific innate immune cell subsets, including dendritic cells, monocytes, and natural killer (NK) cells, have been implicated in post-COVID.

Dendritic cells (DCs) are key players in both innate and adaptive immunity, serving as potent antigen-presenting cells (APCs) crucial for orchestrating immune responses as well as recently being implicated in the maintenance of (intestinal) immune homeostasis (40, 47–49). There are different DC subtypes (type 1 and 2) that exhibit unique functions and responses to viral infections, Reductions in DC numbers, particularly conventional type 1 dendritic cells (cDC1s), have been observed in severe acute COVID-19 and post-COVID in both adults and children (40, 50, 51). This reduction may have implications for antigen presentation and orchestrating an effective adaptive immune response (40, 50, 51). During severe acute COVID-19, circulating plasmacytoid dendritic cells (pDC) populations are significantly depleted (52, 53), which may impair early viral control since pDCs are primary producers of type I interferons (47). Hopkins et al. found that while CD86 expression on pDCs was significantly increased at the point of hospital admission, it returned to a level comparable to healthy controls by two weeks and remained so for the 6–7 month follow-up period (52).

Several studies have linked monocyte subtypes and associated plasma proteins to persistent symptoms following SARS-CoV-2 infection (54–56). One subtype, non-classical monocytes (CD14^low^CD16^high^), exhibits heightened levels in some post-COVID patients, particularly those with increased fatigue severity (51, 52, 57, 58). Additionally, a higher frequency of activated (or intermediate) CD14^+^CD16^+^ monocytes (58, 59) and CD38^+^HLA-DR^+^ activated myeloid cells (including monocytes and pDCs) has been reported in some post-COVID patients (46, 52). Phetsouphanh et al. proposed that these findings may indicate persistent antigen presentation and ^T-cell^ activation (46). This is supported by other studies reporting alterations in monocyte populations in post-COVID patients, including a shift towards CD33-expressing classical monocytes (56). Elevated levels of interleukin-1 receptor type II (IL1R2; expressed by monocytes and macrophages), along with elevated MATN2, CSF3, and IL-6 (all known promoters of inflammation and monocyte activation), have also been observed (55). Furthermore, upregulation of major histocompatibility complex class II (MHC II) expression on monocytes suggests enhanced antigen-presenting capabilities (51). Collectively, these alterations—from subtype shifts to the expression of inflammatory proteins and activation markers—paint a clear picture of a persistently activated monocyte compartment, strongly suggesting an ongoing response to a lingering stimulus such as a persistent viral reservoir, and a dysregulated immune response.

Two studies in adults and children experiencing severe acute COVID-19 reported depletion of natural killer (NK) cells from the bloodstream and their functional impairment attributed to the presence of high levels of transforming growth factor–β (TGF-β) (50, 60). However, evidence regarding NK cell function in post-COVID is less clear. One study found slightly increased expression of the activation marker NKG2C on NK cells in post-COVID patients, but the functional implications of this finding remain uncertain (61). Other studies are needed to confirm whether these NK cells are functionally exhausted or exhibit enhanced activity (61). A small pilot study reported downregulation of human leukocyte antigen B (HLA-B), a component of MHC class I molecules, in post-COVID patients (39). Conversely, this study found significantly upregulated expression of HLA-E, which specifically interacts with CD94 on NK cells (39). HLA-E has been correlated with reduced immunogenicity via decreased CD107a degranulation of NK cells, potentially protecting cells from NK cell-mediated lysis (39, 62–64). The authors of this pilot study suggest that these findings could indicate reduced NK cell inhibition but increased protection of target T-cells from NK cell-mediated lysis; however, further research is necessary to confirm this interpretation.

The complement system serves as a crucial bridge between the innate and adaptive immune responses, orchestrating a cascade of events that enhance pathogen clearance and modulate adaptive immune cell functions. While essential for immunity, unregulated complement activation can drive systemic inflammation, thrombosis, and tissue damage (65). While direct evidence in PPCC remains limited, a convergence of compelling research in adults now strongly indicates that persistent complement dysregulation is a core pathological feature of post-COVID. Two recent, large studies independently found that patients with post-COVID show a clear signature of ongoing complement activation, specifically involving the classical and alternative pathways (65, 66). This dysregulation is linked to a state of thromboinflammation, with markers of tissue injury and red blood cell lysis being prominent (65). Furthermore, specific sets of complement biomarkers have been identified that are highly predictive of a post-COVID diagnosis in adults (66). Where other studies in adults have reported increased levels of complement C4b and C1qA were noted in some post-COVID patients (51, 55). Increased levels of activators of the alternative complement pathway, such as COLEC12, have also been reported (55). One study suggested this process may be driven in part by antibodies against other viruses like CMV and EBV (65). The direct relevance of this thromboinflammatory signature to PPCC remains to be established. Therefore, investigating the complement pathway and its downstream effects in children with PPCC is a critical research priority. Such research could reveal key mechanisms of the disease and confirm whether the complement system is a viable therapeutic target for these younger patients.

#### 2.1.2 Adaptive immune system

The pediatric systemic immune response is characterized by a more naive state with a higher T-cell receptor (TCR) repertoire diversity, whereas adults display a highly cytotoxic, memory-based immune compartment (50). Additionally, the airway epithelium in children has a higher steady-state expression of interferon (IFN)-response genes, which helps restrict viral spread early on (67).

Alterations in especially CD4^+^ and CD8^+^ T-cell subsets have been observed in post-COVID patients, suggesting an imbalance in adaptive immunity (38, 40, 51). Phetsouphanh et al. hypothesized that the observed decrease in naïve T and B cells may be due to persistent conversion of naïve T-cells into activated states, potentially driven by bystander activation secondary to underlying inflammation or antigen presentation by activated pDCs or monocytes (46). This supports the hypothesis that ongoing antigen-specific immunity in post-COVID may result from a chronic SARS-CoV-2 infection, reactivation of a latent virus, or the presence of a persistent antigen (68).

##### 2.1.2.1 T-cell alterations in post-COVID: a state of chronic activation and dysregulation

The T-cell response in post-COVID conditions is characterized by significant dysregulation, suggesting a state of persistent immune activation that differs from both healthy recovery and the acute phase of infection. While adults typically mount a stronger T-cell response to the spike protein during acute infection compared to children (1, 69), and children show a notable expansion of natural regulatory T-cells (nT_regs_) (70), the long-term sequelae point to a sustained imbalance in adaptive immunity (**Box 2**).

Box 2 **T-cell alterations in post-COVID.**

Various studies have looked into the role and presence or absence of T-cell subsets in post-COVID patients. In summary we find that post-COVID individuals exhibit significant alterations in T-cell subsets compared to healthy controls. These alterations include decreased counts of naïve T-cells, central memory CD4^+^ T-cells, and effector memory CD8^+^ T-cells, along with increased levels of T_h_2 cells, T follicular helper (T_fh_) cells, and cytotoxic CD8^+^ T-cells.

These changes suggest that chronic activation of naïve T-cells leads to their depletion and a corresponding increase in the now activated CD4^+^ and CD8^+^ T-cells. This activation is initiated by APC cells, which in post-COVID are most likely monocytes as opposed to cDCs based on decreased cDC and increased monocyte count. The increased T-helper cell count supports the theory of an active T-cell mediated immune reaction. The low level of central and effector memory cells could be explained by the infection not yet being resolved, or the exhaustion markers found various T-cell subsets also hints at chronic viral antigen stimulation.

##### 2.1.2.2 Shifts in T-cell populations and signatures of exhaustion

A primary feature of this dysregulation is the alteration of T-cell subsets (51, 71–74), consistent with chronic activation driving the differentiation of naïve cells. Studies report a decrease in the pool of naïve CD4^+^ and CD8^+^ T-cells (20, 51, 74), although some findings suggest these changes may be specific to certain subsets (e.g., CD127_low_TIM-3^-^CCR7^+^CD45RA^+^CD27^+^ T-cells) rather than a universal depletion (46, 51). This ongoing differentiation impacts the memory compartment, with reports of reduced CD4^+^ central memory cells and certain CD8^+^ effector memory subsets (T_EM_ and T_EMRA_) (51, 75). This could indicate that memory cells are being pushed towards terminal differentiation or are migrating into tissues as Tissue-Resident Memory cells (TRMs). In patients with gastrointestinal symptoms, this process is correlated with an accumulation of cytotoxic CD4^+^ and CD8^+^ T-cells (46, 76–78).

This state of constant stimulation often leads to T-cell exhaustion, a state of functional impairment marked by the elevated expression of inhibitory receptors like PD-1, TIM-3, and CTLA-4 on CD4^+^ and CD8^+^ T-cells (38, 46, 51, 56, 71–74, 79). Evidence for exhaustion, however, is complex. While some studies find elevated PD-1 on central memory T-cells (72), others do not report significant increases in exhausted (PD-1^+^TIM-3^+^) subsets in their cohorts (51). This exhaustion may also be tissue-specific, as an exhausted TIGIT^+^CD8^+^ T-cell subset was found elevated in the cerebrospinal fluid but not the blood of some individuals (38, 51). This exhausted state may also impair immune regulation, as suggested by an inverse correlation between exhausted CD8^+^ T-cells and SARS-CoV-2-specific regulatory T-cells (T_regs_) (38).

##### 2.1.2.3 Functional consequences and altered antigen-specific responses

Functionally, this dysregulation manifests as altered cytokine production and skewed T-cell responses. Some patients exhibit an increase in IL-4- and IL-6-secreting CD4^+^ T-cells (a Th2-skewed response) and elevated levels of T follicular helper (T_fh_) cells, which are critical for directing antibody responses (38, 40, 51, 80, 81). A key finding from another study, Krishna et al., is the persistent, spontaneous release of IFN-γ from CD8^+^ T-cells, which occurs without direct *ex vivo* stimulation and is dependent on antigen presentation by CD14^+^ monocytes (76). Symptom improvement in these patients correlated directly with a decrease in this spontaneous IFN-γ production, providing a strong link between ongoing, antigen-driven T-cell activity and PCC (76).

The response to specific SARS-CoV-2 antigens is also complex. While one study suggested a decreased and more rapidly declining response from nucleocapsid-specific CD8^+^ T-cells (69), others have reported increased numbers or activity of cytotoxic CD8^+^ T-cells (46, 76, 77). Another study by Rodriguez et al., found an inverse correlation between the expansion of SARS-CoV-2-specific memory CD8^+^ T-cells and the level of spike-specific IgG antibodies (56). The authors hypothesized that this “restrained” T-cell response might permit viral persistence, leading to the chronic antigen exposure that drives high antibody levels (56). The wide variability in these findings may stem from different underlying disease mechanisms across patient phenotypes, with bystander activation from cytokines also proposed as a potential driver of the widespread T-cell exhaustion observed (20).

##### 2.1.2.4 B-cells

Fewer studies report on B-cell population in post COVID. One study reported an Increase in effector B cells, as well as specific subsets such as CD86_hi_HLA-DR_hi_ and double-negative (IgD^-^CD27^-^CD24^-^CD38^-^) B cells in adults with post-COVID (51). The absence of naïve CD127_low_TIM-3^-^CD38_low_CD27^-^IgD^+^ B cells has also been reported in some post-COVID patients (46). Furthermore, in some post-COVID patients, RBD-specific antibody titers did not positively correlate with total SARS-CoV-2-specific CD4^+^, CD8^+^, or CD4^+^ follicular helper ^T-cell^s, unlike in recovered individuals, suggesting a potentially uncoordinated humoral and cell-mediated immune response (38).

During SARS-CoV-2 infection, children demonstrate a predominant expansion of immature B lymphocytes and natural regulatory T cells (Tregs) (50). However, in severe cases, the virus can disrupt typical priming. Severe COVID-19 can induce a transient, naive-derived antibody-secreting B cell compartment that is enriched in autoreactive potential (82).

##### 2.1.2.5 Anti-viral antigen antibodies

Findings regarding antibody responses in post-COVID patients are divergent, with evidence for immunity to Spike (S) and Nucleocapsid (N) antigens that may be equal to, reduced, or enhanced compared to individuals without persistent symptoms.

Initial studies suggested that low or absent SARS-CoV-2 antibody production, particularly lower anti-S levels, during the acute infectious stage, might predict post-COVID development whereby a lack of seroconversion could hint at an ineffective acute response to the virus (83, 84). These results were corroborated by a study reporting an inverse correlation between anti-N antibody levels and the likelihood of persistent symptoms (85). However, later studies reported that, even when controlling for vaccination status with matched controls, levels of spike- and S1-specific IgG were higher in some post-COVID patients (51, 78). Furthermore, an increased presence of IgG against the SARS-CoV-2 nucleocapsid protein was associated with the development of post-COVID neurological manifestations in some individuals (51, 78). These later findings have been interpreted as potentially consistent with persistent viral antigens (51). Total receptor binding domain (RBD)-specific antibody titers were found to be increased more than twofold in some post-COVID patients compared to recovered individuals, and have been proposed as a potential sensitive marker of severe post-COVID, possibly resulting from chronic antigen stimulation and viral persistence (38, 56).

#### 2.1.3 Auto-antibodies

COVID-19, the “infectome paradigm of autoimmunity” (86) has been linked to autoimmunity, potentially through molecular mimicry between viral and human antigens (86, 87). While the role of autoantibodies (AABs) in the long-term effects of COVID-19 is still under investigation, Altmann et al. demonstrated that they are frequently detected during the acute phase of infection, especially in severe cases (20). Evidence for a triggered autoimmune response includes the cross-reactivity of anti-SARS-CoV-2 antibodies with human autoantigens and the presence of AABs in patients’ sera that are known to cross-react with the virus (86, 88).

Despite the high prevalence of AABs during acute COVID-19, their association with post-COVID conditions (PCC) is not consistently reported. Some studies have shown correlations, such as the link between anti-SARS-CoV-2 antibodies and neutralizing autoantibodies against type I interferons (78). Additionally, transferring IgG from patients with PCC has been shown to induce symptoms in mice (89). Other research has identified latent autoimmunity in 83% of patients with post-COVID syndrome and elevated levels of AABs against targets like ACE2 and various receptors, suggesting a role in chronic damage (35, 90). For example, the presence of antinuclear autoantibodies (ANAs) three months post-infection has been predictive of systemic symptoms at 12 months (91). Specific AABs have also been linked to post-COVID endotypes, such as anti-IFNα2 autoantibodies with pulmonary disease (78) and functional AABs against G protein-coupled receptors (GPCRs) (92).

However, other large studies have not found a significant association between AABs and post-acute sequelae of COVID-19 (51, 93). The divergent findings on the role of autoantibodies in post-COVID are largely explained by differences in the specific targets tested and the clinical characteristics of study cohorts, particularly the severity of the initial infection. This suggests that while autoantibody-driven pathology may be a key factor in patients recovering from severe COVID-19, other mechanisms, such as persistent cytokine elevation, may be more prominent in most post-COVID cases that arise from mild illness. Nonetheless, the increased incidence of new-onset autoimmune diseases—such as type 1 diabetes, rheumatoid arthritis, and lupus—following COVID-19 infection underscores the potential for autoimmunity in PCC (20, 86).

### 2.2 Persistent viral reservoir

The majority of individuals infected with SARS-CoV-2 clear the live virus within days to a few weeks after infection, to the point that it is no longer detectable in the respiratory system by standard methods (68). However, some evidence suggests that persistent viral presence may contribute to post-COVID conditions in some individuals (20). Viral persistence in ‘sanctuary tissues’ has been demonstrated with other viruses, such as CMV, EBV, Herpes viruses, as well as Ebola (94–96).

While SARS-CoV-2 primarily infects respiratory epithelium, various other cell types throughout the body express ACE2 and TMPRSS2 (co)receptors. These include alveolar macrophages, renal tubular cells, gastrointestinal epithelia in the ileum, colon and rectum, esophageal keratinocytes, liver cholangiocytes, and neurons, among others (35, 45, 68, 86). SARS-CoV-2 RNAs and proteins have been detected in the cardiovascular system, stomach, intestines, breast, skin, thyroid, brain, lung tissue, renal tissue, plasma, and urine long after initial infection (35, 97–99). In a comprehensive meta-analysis, Cevik et al. demonstrated that SARS-CoV-2 viral shedding can persist for a prolonged period, reporting a maximum duration of 83 days in the upper respiratory tract, 59 days in the lower respiratory tract, 126 days in stool samples, and 60 days in serum samples (100). SARS-CoV-2 has been shown, in some cases, to establish long-term, non-productive persistent infections in various tissues and cell types (16, 97, 101, 102). Evidence for viral persistence includes the detection of SARS-CoV-2 components in various tissues—from biopsies of living patients to post-mortem samples—using highly sensitive methods like digital droplet PCR (ddPCR), immunofluorescence, electron microscopy, and immunohistochemistry (97–99).

Recently, a significant association between viral RNA persistence and the development of post-COVID symptoms has been demonstrated (97, 103). For example, Ghafari et al. found that prolonged detection of high-titer viral RNA in respiratory swabs was associated with a more than 50% higher odds of self-reporting post-COVID (103). Going further, Zuo et al. detected persistent viral RNA in a wide range of solid tissues from living patients—including the liver, kidney, stomach, intestines, brain, and lungs—and also found a significant correlation with post-COVID symptoms (97). The presence of viral components in these tissues has been hypothesized to contribute to chronic inflammation and multiple systemic manifestations, although this remains hypothetical and no direct causal link has yet been established.

The establishment of SARS-CoV-2 persistent reservoirs has been associated with factors including immunosuppression, reduced expression of ribosomal proteins, downregulation of immune response genes, dysregulation of genes related to complement, coagulation, and cholesterol metabolism, and the potential for integration of selected SARS-CoV-2 sequences (16, 97, 104). Cell-to-cell contact spreading, a particle-independent mechanism, may contribute to immune evasion (105) as demonstrated with *in vitro* experiments (16, 106). SARS-CoV-2 may persist possibly through modulation of the host transcriptional program, as suggested by studies in lung tissue of patients with viral persistence, or by cell-to-cell fusion (16, 97). It is hypothesized that a concurrence of molecular and immunological events allows the virus to influence the host transcriptional program, enabling viral replication without killing the host^T-cell^ and simultaneously evading the immune response (16). Additionally, the Spike protein has been shown to be shed by host^t-cells^ via extracellular vesicles (EVs) and can be distributed via the circulatory system (107, 108).

The gastrointestinal tract is the most extensively studied potential sanctuary site for residual viral depots. This is due to the relative ease of access for biopsies, the usefulness of fecal samples as a proxy, and the high expression of ACE2 on cells within the GI tract (20). Prolonged fecal viral shedding has been associated with persistent gastrointestinal symptoms such as diarrhea, nausea, vomiting, or abdominal pain in both adults and children (11, 109–112). Viral antigens were detected in adult stool samples for up to 12 months after SARS-CoV-2 infection, and in the gut epithelium for up to seven months after initial infection, regardless of long-COVID status (20, 35).

A compromised humoral immune response has been linked to viral persistence in the gastrointestinal tract. One study found that patients with viral rebounds produced significantly lower titers of receptor-binding domain (RBD)-specific IgA and IgG antibodies; for example, fewer than 45% of these patients generated high levels of IgG, compared to over 90% of patients who cleared the virus (113). Furthermore, this antibody response was slower, with some patients showing delayed IgA generation until week 7 post-infection (113).

In contrast, other research provides evidence supporting ongoing antigenic stimulation. One study reported that while neutralizing antibody levels in the blood decreased over six months, the number of memory B cells remained stable, and their B cell receptors (BCRs) accumulated somatic hypermutations, indicating continued evolution (114). To investigate the source of this stimulus, the same study analyzed intestinal biopsies from 14 asymptomatic individuals approximately 4 months after infection. SARS-CoV-2 antigen and RNA were detected in the small bowel of 7 of these participants, even when their respiratory swabs were negative (114). However, this viral persistence in the gut did not appear to elicit any local inflammatory infiltrate, raising questions about the immunogenicity of this potential reservoir and the source of the ongoing systemic antigenic stimulus (20).

However, evidence of a persistent immune stimulus is also found in the ^T-cell^ dynamics of post-COVID patients with gastrointestinal (GI) complaints. A multi-omic study demonstrated that at 2–3 months post-infection, these patients uniquely exhibited a significant expansion of newly emerging cytotoxic CD8^+^ and CD4^+ T-cell^ clonotypes when compared to patients without GI symptoms (78). This continued generation of new effector ^T-cells^ long after the acute phase strongly suggests a lingering antigenic source, potentially in the GI tract driving these responses (78). Furthermore, a study in the related pediatric condition MIS-C demonstrated that prolonged SARS-CoV-2 presence in the gastrointestinal tract was associated with a loss of gut barrier integrity and subsequent circulation of viral spike antigen in the blood, which was proposed to drive hyperinflammation (115). The exact nature of this stimulus and its direct link to symptoms requires further investigation.

Although few studies have specifically investigated viral persistence in children, one hypothesis suggests it could be more common as a result of prioritization of stature growth over virus-eradicating systemic inflammatory responses, as a mechanism to conserve limited resources (14, 56, 116). However, this remains a theoretical concept, and further research is needed to confirm its validity (117).

Viral persistence could potentially result from ineffective, decreased, or exhausted effector immune cells in sanctuary tissues, leading to incomplete viral clearance after the acute infection phase (75, 80, 97). Post-COVID patients with worse quality-of-life and more severe cognitive symptoms had reduced CNS effector molecule expression in memory ^T-cells^, suggesting a possible deficiency of these cells (80). The inverse correlation between elevated IgG responses and the frequency of clonally expanded memory CD8^+ T-cells^ in some post-COVID patients also raises the possibility of viral persistence (56). A more rapid decrease of N-specific IFN-γ^+^/CD107a^+^ and IFN-γ^+^/CD8^+ T-cells^ in some post-COVID patients has been interpreted as potentially resulting from ^T-cell^ exhaustion due to persistent antigen stimulation, suggesting a possible dysfunctional ^T-cell^ response (69, 118). On the contrary, elevated frequencies of IFN-γ- and TNF-α-producing SARS-CoV-2-specific ^T-cells^ in individuals with pulmonary post-COVID symptoms were found to be associated with increased systemic inflammation and decreased lung function, also suggesting a potential pathogenic role for SARS-CoV-2-specific ^T-cells^ via the production of inflammatory cytokines (119).

Nevertheless, the precise mechanisms by which persistent viral presence, if present, might contribute to post-COVID symptoms remain unclear, and warrant further investigations (68).

### 2.3 Reactivation of latent viruses

Reactivation of latent viruses has been associated with both acute and post-COVID conditions, as well as with the severity and duration of symptoms (120, 121). Specifically, fatigue, neurocognitive symptoms, and a gastrointestinal symptom profile have been linked to viral reactivation, and Epstein-Barr virus (EBV) reactivation, in particular, has been identified as a potential risk factor for post-COVID development (3, 78, 86, 121, 122). SARS-CoV-2 infection has also shown to co-occur with reactivation of other herpesviruses, including cytomegalovirus (CMV), and herpes simplex virus (HSV) types 1, 2, and 3 (68, 86). EBV and human herpesvirus 6 (HHV-6) are among the most common immunotropic viruses that establish latency after infection (78, 121, 122). Approximately 90% of the human population harbors EBV (123, 124), with latent viral persistence primarily within memory B cell reservoirs (86). Reduced general and local immunity including COVID-19, may trigger EBV reactivation (121). In non-COVID settings, various mechanisms of EBV reactivation have been identified, including host/viral mRNAs, other viral genes, histone modifications, reactive oxygen species, cellular stress responses, cell transcription factors binding to viral promoters, cytokine-mediated reactions, and loss of immune control (68). Additionally, EBV lytic replication can induce ACE2 expression on epithelial cells, potentially facilitating SARS-CoV-2 entry and potentially contributing to increased viral load and symptom severity, as suggested by previous studies (125, 126).

In particular EBV reactivation has been associated with PCC, especially fatigue and neurocognitive dysfunction in some patients with post-COVID (122, 124). Other studies have found elevated levels of antibodies to several herpesvirus antigens, including EBV capsid and receptor proteins, and varicella-zoster virus (VZV) glycoprotein (51), though ruled out this was due to acute viral infections (51). It remains unclear whether there is a direct pathogenic effect of latent virus reactivation or whether a transient phase of viral reactivation during acute SARS-CoV-2 infection induces additional inflammatory immune responses that contribute to the development, continuation, or exacerbation of post-COVID conditions.

Further research is needed, especially in children, to determine the extent to which latent virus reactivation contributes to the pathogenesis of post-COVID conditions and whether targeting these reactivated viruses could offer therapeutic benefit.

### 2.4 Vascular (endothelial) inflammation

COVID-19-associated hyperinflammation can cause endothelial dysfunction and a hypercoagulable state, which, while most studied in acute disease, are also implicated in post-COVID conditions (35, 40, 127–130). Vascular changes may play a role in organ-specific post-COVID symptoms, including pulmonary complications such as dyspnea, neurovascular issues like brain fog and headache, and cardiorespiratory complaints such as chest pain and pulmonary hypoperfusion, as well as fatigue and musculoskeletal pain (35, 131–134). Mechanisms of thrombo-inflammation include endothelial injury, complement activation, platelet activation and platelet–leukocyte interactions, neutrophil extracellular trap (NET) formation, release of pro-inflammatory cytokines, disruption of normal coagulant pathways, and hypoxia (**Box 3**) (20, 73).

Box 3 **Vascular & coagulatory pathophysiology in post-COVID conditions.**

Core Pathology: COVID-19-associated hyperinflammation drives endothelial dysfunction and a hypercoagulable state. While prominent in acute disease, these processes persist and are implicated in post-COVID conditions^35,40,125–128^.

Clinical Manifestations:

Vascular changes contribute to organ-specific symptoms, including^35,129–132^:

• Pulmonary: Dyspnea and pulmonary hypoperfusion.

• Neurovascular: Brain fog and headache.

• Cardiorespiratory: Chest pain.

• Systemic: Fatigue and musculoskeletal pain.

Mechanisms of Thrombo-inflammation: The underlying drivers include^20,72^:

• Endothelial injury and hypoxia.

• Complement activation.

• Platelet activation and platelet–leukocyte interactions.

• Neutrophil extracellular trap (NET) formation.

• Pro-inflammatory cytokine release.

• Disruption of normal coagulant pathways.

During acute COVID-19, SARS-CoV-2 can contribute to the breakdown of the endothelial–epithelial barrier leading to the expression of various endothelial proteins that promote a procoagulant environment and thrombus formation (20, 135). This may contribute to long-term changes in the vessels themselves, with alterations in size, stiffness, and vascular density observed in some patients with post-COVID (136–138). In some post-COVID patients, this state of endothelial dysfunction persists, with studies showing elevated levels of proteins such as endothelin-1 (ET-1) and plasminogen activator inhibitor 1 (PAI-1), and reduced levels of ADAMTS13, potentially contributing to persistent clot formation (20, 139). Pulmonary vascular microthrombosis and macrothrombosis have been observed in 20-30% of patients with acute COVID-19, and the persistence of this thrombotic endotheliitis has been proposed as a potential driver of post-COVID (73, 140). Additionally, large anomalous (amyloid) deposits (microclots), found in both acute and post-COVID conditions, are resistant to fibrinolysis (141, 142). In children with PCC, a distinct pro-angiogenic signature, characterized by elevated levels of factors like CXCL1, CXCL5, CXCL6, and OSM, has also been identified, supporting the role of vascular dysfunction in the pediatric manifestation of the condition (143).

Post-COVID patients, both adults and children, have demonstrated increased levels of D-dimer, factor VIII, IL-6, von Willebrand factor (vWF), and vWF propeptide, signs of basement membrane exposure and coagulation cascade activation (20, 127, 132, 144–146). Additionally, platelet hyperactivation, driven by viral inflammation can result in hypercoagulability and can contribute to thrombocytopenia, and was found to be dependent on a type I interferon (IFN) signaling pathway (147).

This ongoing prothrombotic state in post-COVID is thought to involve a cycle where activated platelets, complement factors, and (antiphospholipid) autoantibodies activate neutrophils to release Neutrophil Extracellular Traps (NETs), which are themselves highly prothrombotic (20). While high levels of the necessary antibodies have been clearly demonstrated in patients with acute COVID-19, their precise prevalence and pathogenic role in establishing post-COVID is still an area of active investigation (20).

Elevated expression levels of proteins correlated with apoptosis and epithelial injury have been reported in post-COVID patients (3, 148). Specifically, the angiogenesis markers ANG1 and P-selectin have demonstrated high sensitivity and specificity for predicting post-COVID status in some studies (149). P-selectin, a sensitive marker of platelet activation, was elevated in both post-COVID children and adults compared to healthy controls (150, 151). Platelet activation could be a consequence of a persistent viral reservoir (potentially including platelets themselves) or, more generally, the result of endothelial inflammation secondary to chronic immune dysfunction (65, 97, 107, 150–152).

### 2.5 Neuroinflammation, injury, and dysfunction

Neurological and cognitive symptoms—including cognitive impairment, brain fog, and autonomic dysfunction—are among the most frequently reported sequelae in both children and adults with post-COVID conditions (3, 153). The underlying pathophysiology is multifactorial, with proposed mechanisms including direct neuronal injury, damage to the neurovasculature, neuroinflammation, and viral persistence (3, 35, 154, 155).

Evidence supports SARS-CoV-2 as a neurotropic virus capable of infecting nerve cells and persisting in the nervous system for months, where it is associated with neuroinflammation, hypoxia, and micro-hemorrhages (33, 156–161). This neuroinflammation (156, 162, 163), driven by mechanisms like microglial activation and autoimmune reactions, can lead to significant pathology (35, 157, 164). Autopsy studies reveal SARS-CoV-2-associated changes in brain parenchyma and vessels, possibly due to blood-brain barrier disruption (165, 166). In children, post-COVID neuroinflammatory diseases such as encephalopathy and acute disseminated encephalomyelitis have been reported (167). Additionally, NFASC, Spondin-1, and IDS were all found to be upregulated, along with glial maturation factors (GFM-B and CFM-G), suggesting a potential neuroinflammatory response to tissue damage through upregulating neuronal growth and leukocyte infiltration (39, 55). Furthermore, some post-COVID neurological complications may be exacerbated by chronic, age-related “inflammaging” and Alzheimer’s-like pathology, including inflammasome activation through TGF-β signaling, and elevated levels of the complement component C1qA (55, 71, 73, 168).

#### 2.5.1 Brain damage

Studies have identified biomarkers of this pathology, though findings are not always consistent. Markers of cerebral injury such as neurofilament light chain (NfL), GFAP, and p-tau-181 have been found to be elevated in post-COVID patients and associated with disease progression (169–171). However, other studies found only IL-6 to be significantly correlated with neurological sequelae (172). Certain PCC patients with decreased cognitive function were found to have perturbation of specific miRNAs that have previously been associated in other neurodegenerative pathologies, and have been linked to pro-inflammatory target genes regulating cytokines such as IL-6, as well as IL-8, IL-17, NF-κB, IFN-λ, and IFN-β, linking systemic inflammation to cognitive function (171). Interestingly, extracellular vesicles derived from neurons have been reported to contain components relevant to potential neural damage and have been suggested as a candidate for monitoring the neuropsychiatric manifestations of post-COVID (160, 172).

#### 2.5.2 Neuronal dysfunction

Beyond direct inflammation and damage, post-COVID is also marked by signs of broader neuronal dysfunction. These include the presence of toxic, self-assembling amyloid peptides from the SARS-CoV-2 proteome; brain and brainstem hypometabolism linked to “brain fog”; and myelin loss with microglial reactivity similar to that seen in “chemo-brain” (155, 157, 173–175). Additionally, disruptions in metabolic pathways, such as tetrahydrobiopterin deficiencies, may alter key neurotransmitter systems like serotonin and dopamine, potentially affecting memory and other cognitive functions via mechanisms that could involve the vagus nerve (147, 176). Interestingly, tetrahydrobiopterins play a major role in converting amino acids such as phenylalanine, tyrosine, and tryptophan into precursors of dopamine and serotonin, key monoamine neurotransmitters that have been implicated in the broader metabolic dysfunction observed in some PCC patients (see relevant section) (176).

In summary, viral persistence and the resulting neuroinflammation appear to be critical mechanisms contributing to the neurological sequelae of post-COVID. This inflammation, potentially in concert with autoimmune reactions and metabolic disturbances, can lead to direct neuronal damage and widespread dysfunction.

### 2.6 Metabolic/mitochondrial dysfunction

#### 2.6.1 Metabolic dysfunction

Musculoskeletal pain, cognitive impairment, persistent headaches, and constitutional symptoms such as fatigue, malaise, and post-exertional malaise (PEM) have all been associated with metabolic and/or mitochondrial dysfunction (11, 177). SARS-CoV-2 can directly interfere with cellular energy production, as 15% of the bound host polypeptides are mitochondrial (35, 178–182). During acute infection, the virus alters the mitochondrial structure, inhibits oxidative phosphorylation (OXPHOS), increase mitochondrial reactive oxygen species (mROS) production, shift metabolism towards glycolysis, trigger an integrated stress response and a broader inflammatory cascade (178). Critically, this suppression of mitochondrial genes and OXPHOS function may be irreversible and persist long after the virus is cleared, contributing to the chronic symptoms of post-COVID (37, 178). In children with post-COVID, evidence of this is seen in salivary biomarkers showing a clear imbalance towards oxidative stress (183).

##### 2.6.1.1 Post exertional malaise

The clinical consequences of this energy deficit are widespread. In patients with PEM, this is observed directly in skeletal muscle, which shows a higher proportion of highly fatigable glycolytic fibers, significantly lower OXPHOS capacity, decreased tricarboxylic acid (TCA) cycle metabolites, and infiltration of amyloid-containing deposits (177, 184). These abnormalities worsened significantly after exertion, along with an exacerbated tissue damage response characterized by small atrophic fibers and focal necrosis (177, 184). Inflammatory infiltration has also been documented, with an increased presence of CD68^+^ macrophages and an attenuated CD3^+ T-cell^ response (177). While inflammation may trigger abnormal catabolic pathways and resultant muscle breakdown, no significant difference in breakdown products such as creatinine or creatine kinase was observed (177, 185). It has been therefore hypothesized that the OXPHOS disturbance observed in PEM may be a residual effect from the acute infectious period, resulting from SARS-CoV-2 peptide binding and inhibition of mitochondrial transcription (178).

##### 2.6.1.2 Pancreas damage

Beyond muscle, the dysfunction manifests as impaired insulin/IGF-1 signaling, which can result in insulin resistance, hyperlipidemia, and new-onset diabetes (33, 186–189). for example, diabetic ketoacidosis (DKA) has been observed in patients without known diabetes mellitus weeks to months after resolution of COVID-19 symptoms (190). Additionally, in a cohort of hospitalized but recovered patients without pre-existing diabetes, 46% developed hyperglycemia 2–5 months after initial infection (191, 192). However, at 12 months, less than 20% of patients initially diagnosed with new-onset diabetes still exhibited hyperglycemia, a recovery that raises questions about the long-term persistence of these metabolic changes (192). The mechanism may involve direct virus induced damage to the pancreas via its ACE2 receptors, leading to impaired beta-cell function (33, 35, 157, 193).

##### 2.6.1.3 Cortisol

Furthermore, observations of low cortisol levels in some patients without a compensatory rise in ACTH suggests a potential dysregulation of the hypothalamic-pituitary-adrenal (HPA) axis, adding another layer to the systemic metabolic disruption (51, 78). Those with low cortisol levels tended to be patients presenting with persistent neurological or respiratory symptoms (51, 78).Kynurenine pathway.

Specific metabolic pathways are also significantly altered, particularly those governing the essential amino acid tryptophan (36, 147, 194–199). In the inflammatory environment of post-COVID, tryptophan metabolism is often shunted away from serotonin production and towards the kynurenine pathway (KP) (36, 196, 197). This shift is driven by inflammatory cytokines (e.g., IFN-γ, TNFα) that induce the enzyme indoleamine 2,3-dioxygenase 1 (IDO1), leading to the production of neurotoxic metabolites like quinolinic acid, which are associated with cognitive impairment (36, 194, 200). IDO1 has additionally been shown to engage mechanisms of immune tolerance, potentially possessing immunosuppressive functions through decreasing effector ^T-cell^s and NK cells, and upregulating regulatory ^T-cell^s (T_reg_ cells) (36, 201). In some patients, expression of a related enzyme, IDO2, in monocytes and lymphocytes has been directly correlated with mitochondrial dysfunction. The promotor region for the activating enzyme of IDO2 contains several sites for NF-κB binding, suggesting that inflammation is a key factor modulating its expression (202, 203), and linking these two major axes of post-COVID pathophysiology (36).

##### 2.6.1.4 Serotonin

**Figure 2:**
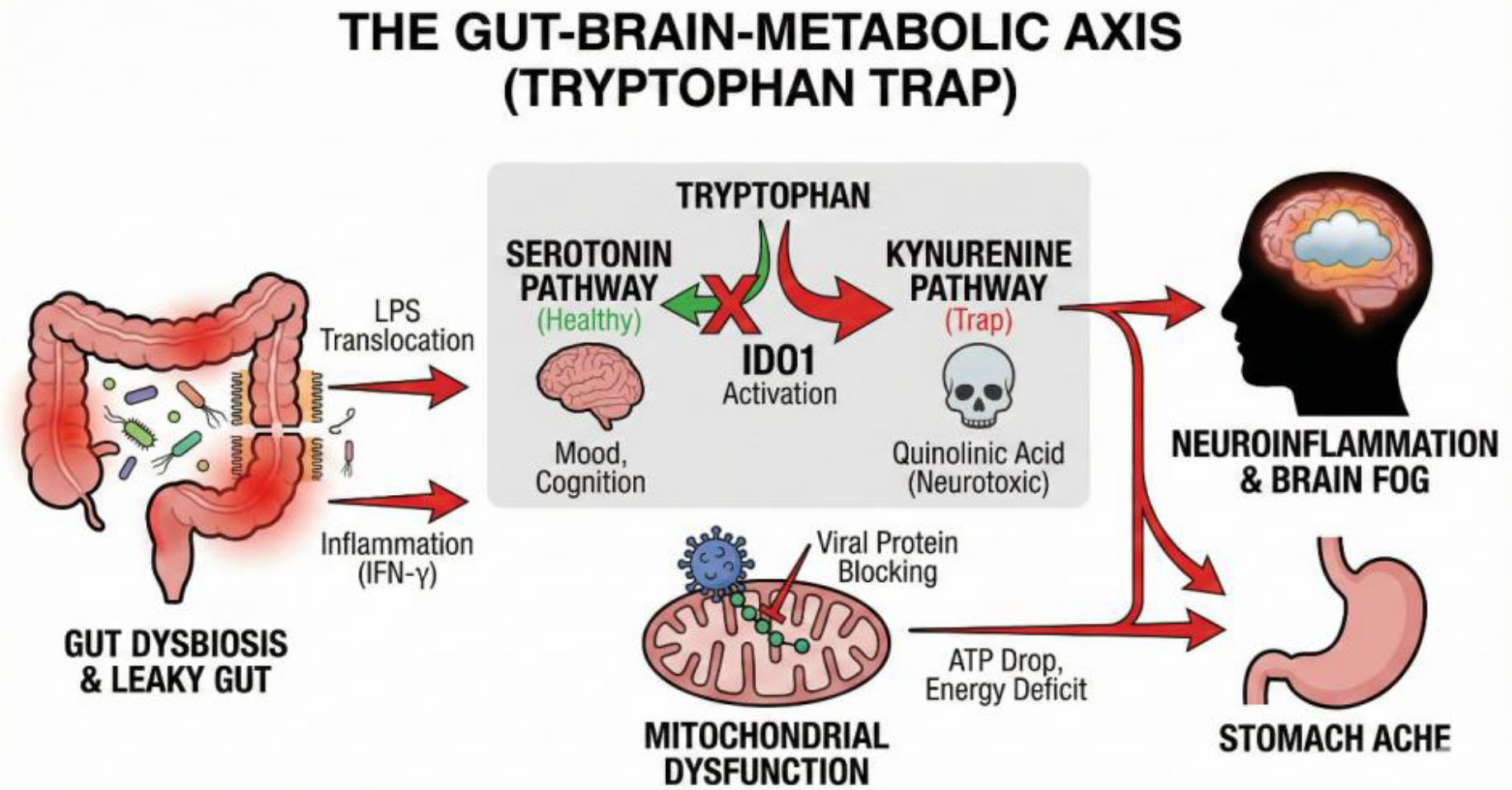
The Gut-Brain-Metabolic Axis (tryptophan trap).

The diversion of tryptophan to the kynurenine pathway could be a major factor in the serotonin depletion observed in some post-COVID patients, particularly those with neurocognitive symptoms (36, 147). This depletion is compounded by several other mechanisms: viral inflammation (via type I IFN) can reduce intestinal absorption of tryptophan, while platelet hyperactivation and thrombocytopenia impair serotonin’s primary storage and transport system (**Figure 2**) (36, 147). The dual role of ACE2 as both a viral receptor and a mediator of intestinal tryptophan absorption is noteworthy, considering the possibility of downregulation following receptor internalization, potentially augmenting the effects of IFN and contributing to the reduction in serotonin (147, 204, 205). As circulating serotonin cannot cross the blood-brain barrier, the influence of peripheral viral inflammation caused serotonin depletion on brain function is thought to be indirect, occurring via afferent sensory neurons; impairment of one such neuron, the vagus nerve, has been proposed as a contributing factor (36, 206, 207). Furthermore, deficiencies in tetrahydrobiopterin—a necessary cofactor for producing both serotonin and dopamine—may also contribute to this neurotransmitter imbalance (3, 176). It should be noted that, while a correlation exists, a direct causal link between these mechanisms and serotonin depletion in all post-COVID patients has not been definitively established.

In summary, evidence strongly suggests that significant metabolic and mitochondrial dysfunctions are potential drivers of post-COVID symptoms. Direct viral disruption of mitochondrial OXPHOS can trigger persistent energy deficits and inflammatory cascades. These disruptions, along with profound imbalances in the kynurenine and serotonin pathways, contribute to a complex interplay of mitochondrial impairment, chronic inflammation, and neurotransmitter deficiencies that can explain many features of the condition.

### 2.7 Microbiome dysbiosis

The final hypothesis to be discussed concerns dysbiosis of various microbiota, particularly those of the gut and lungs. These microbial communities have been shown to have a potentially pivotal role in the pathogenesis, clinical severity, and outcome of acute COVID-19, and have also been implicated in neurocognitive and respiratory post-COVID symptoms, and even in the potential for treatment of post-COVID conditions (208–212). In the acute phase, gastrointestinal symptoms of SARS-CoV-2 infection are common, as the GI tract is a known site of SARS-CoV-2 tropism (213, 214). A meta-analysis found a pooled prevalence of any GI symptom in children with acute COVID of 16.3%, and pooled prevalences for more specific symptoms of abdominal pain, nausea or vomiting, and diarrhea of 8.8%, 11.0%, and 11.1%, respectively (215). An acute SARS-CoV-2 infection has also demonstrated the potential to alter the gut microbiota, enrich certain opportunistic pathogens, deplete various beneficial commensals, and trigger an immunogenic reaction (86, 216, 217). Dendritic cells, as discussed previously, play a role in intestinal immune homeostasis, and likely interact, directly or indirectly, with the gut microbiota to achieve this (40, 47–49). Additionally, SARS-CoV-2 presence in the gut has been linked to increased intestinal permeability (“leaky gut syndrome”) through several proposed mechanisms: microbiota dysbiosis, overproduction of endotoxins secondary to angiotensin accumulation, and activation of monocytes and neutrophils, leading to the production of S100A12 and damage to tight junctions (86, 87, 218). The interaction between the intestinal microbiome and host mitochondria has also been established, with host-produced reactive oxygen species (ROS) indirectly regulating the intestinal epithelial barrier, and microbiota, in turn, influencing mitochondrial function and associated respiratory chain and ATP production (219). Furthermore, there is strong evidence linking microbiota dysbiosis to metabolic dysregulation, including alterations in tryptophan catabolism, in a manner that can potentially fuel inflammation and influence disease severity (194). These mechanisms mentioned often lead to increased inflammation, which, in turn, can lead to more gut dysbiosis, perpetuating the vicious circle.

#### 2.7.1 Microbiome dysbiosis and post-COVID

There is substantial evidence that perturbation of the gut and other microbiota is associated with both the induction and disease severity of post-COVID conditions (208). A large, multi-kingdom gut microbiota analysis identified bacterial, fungal, and viral microbiome signatures that were associated with a doubling of risk for the development of post-COVID (209). Interestingly, different microbiome compositions have been associated with various post-COVID endotypes; for example, gut microbiota alterations have been shown to correlate with post-COVID patients with GI symptoms, and have also been linked to respiratory dysfunction even months after hospitalization (35, 209, 220). Persistent alterations of the gut microbiota following recovery from acute infection were characterized by an underrepresentation of commensals known for their immunomodulatory potential (221).

##### 2.7.1.1 Specific presence and abundances perturbations

Emerging evidence suggests that microbiota imbalance can trigger systemic response associated with post-COVID symptoms, often mediated by interactions with metabolic and immune dysregulation. It has been associated with an increase in the expression of various (pro-inflammatory) mediators, including ACE2, TMPRSS2, IFN-γ, IL-17A, IL-6, CRP, lipopolysaccharide-binding protein (LBP), β-glucan, and zonulin (220, 224, 225).

Beyond direct interaction with the innate and adaptive immune systems, various potential pathways and mechanisms of action have been identified. Disruption of tight junctions may lead to leakage of microbial products like LBP, which can stimulate the enteric nervous system and signal to the CNS, potentially contributing to post-COVID neurological symptoms (225). LBP levels have been shown to remain high after COVID-19 hospitalization and have been associated with low-grade inflammation and respiratory dysfunction (220). β-glucan, a fungal cell wall polysaccharide that has been observed to translocate to the plasma of individuals experiencing post-COVID, has also been correlated with higher inflammation and has been linked to NMDA receptor activation (involved in the tryptophan catabolism pathway), potentially leading to increased levels of metabolites with neurotoxic properties (194). It can also directly induce inflammation by binding to myeloid cells and activating spleen tyrosine kinase/nuclear factor-κB (Syk/NF-κB) signaling (194).

Depletion of certain beneficial gut commensals, such as *Bifidobacterium adolescentis*, has also been observed in COVID-19 patients (**Table 1**). *B. adolescentis* is able to suppress lipopolysaccharide (LPS)-induced activation of NF-κB and thus the expression of proinflammatory cytokines (221, 226). The depletion of *B. adolescentis* has therefore been linked to increased anxiety (227). Also, lower levels of butyrate-producing bacteria, such as *Faecalibacterium prausnitzii*, have been correlated with post-COVID conditions (209, 217). Among the bacterial species negatively associated with post-COVID, those that favor butyrate production are of particular interest, potentially offering therapeutic avenues, due to butyrate’s immunoregulatory roles (199, 209). Short-chain fatty acids (SCFAs) like butyrate have been shown to promote intestinal IgA production by enhancing plasma B cell metabolism and differentiation, potentially protecting against mucosal inflammation (228). However, butyrate can also activate IL-10-producing B cells (predominantly regulatory B cells, or B_reg_ cells) through a mechanism involving the AhR receptor (the same receptor implicated in IDO2 activation). This activation is thought to be mediated, in part, by increasing serotonin-derived 5-hydroxyindoleacetic acid (5-HIAA), the main metabolite of serotonin, which then activates AhR^+^ B_reg_ cells (199, 221, 229), linking gut dysbiosis with cognitive dysfunction. Butyrate has also been shown to promote T_reg_ cell generation, potentially contributing to the regulation of systemic inflammation (221, 230, 231). A reduction in butyrate-producing bacteria in the gut could potentially contribute to decreased tryptophan levels through a loss of inflammation modulation and activation of IDO, which, in turn, has been linked to reduced serotonin levels and post-COVID neurocognitive symptoms (36, 147, 232). Dysregulation of the gut microbiota has also been linked to lower serotonin levels, which as discussed may contribute to post-COVID symptoms (147, 198). Interestingly, healthy mice that were transplanted with gut bacteria from post-COVID patients demonstrated both loss of cognitive functioning and impaired lung defenses (223). This finding suggests that the altered gut microbiome itself can transfer different aspects of the post-COVID phenotypes and may therefore play a central upstream role in the process PCC pathogenesis, although the specific mechanisms remain to be elucidated.

**Table 1:** Various increased and decreased microbes that have been associated with post-COVID.

Increased in post-COVID		
Niche	*Microbe*	Reference
Gut	*Ruminococcus gnavus*	(209)
Gut	*Bacteroides vulgatus*	(209)
Gut	*Veillonella*	(209)
Gut	*clostridial* strains	(209)
Gut	Candida glabrata (fungal)	(209)
Gut	Vibrio phage (virome)	(209)
Gut	Klebsiella phage (virome)	(209)
Oral	*Veillonella*	(222)
Oral	*Prevotella*	(222)

Decreased in post-COVID		
Niche	*Microbe*	Reference
Gut	*Faecalibacterium prausnitzii*	(209)
Gut	*Blautia wexlerae*	(209)
Gut	*Bifidobacterium longum*	(209, 223)
Gut	*Brautia*	(209)
Gut	*Roseburia hominis*	(221)
Gut	*Bifidobacterium adolescentis*	(221)
Gut	*Bacteroidetes*	(209)

Protective microbiota have also been shown to promote an IFN signature in lung epithelia, potentially helping to impede early viral replication (233). This crosstalk is bidirectional; for example, IFN-I production in the lungs during influenza infection has been shown to lead to gut microbiota dysbiosis through the depletion of obligate anaerobes (213, 234). In summary, evidence indicates that gut and lung microbiome dysbiosis, triggered or exacerbated by SARS-CoV-2 infection, may play a role in the development and persistence of post-COVID symptoms, but evidence of the direct causation or successful intervention through this route is not conclusive yet. This dysbiosis is characterized by the depletion of beneficial commensals and the overgrowth of opportunistic bacterial, fungal, and viral species. These imbalances can potentially lead to systemic inflammation, increased gut permeability, and immune dysregulation. Furthermore, disrupted microbiome composition may influence neurotransmitter levels (e.g., serotonin) and exacerbate gut-lung axis dysfunction, potentially contributing to neurocognitive and respiratory symptoms observed in post-COVID patients. Given that the microbiome has been shown to be a potent modulator of immunity and inflammation, not only in the acute phase but also with a potential link to post-acute symptomatology, it presents a promising target for therapeutic interventions (213).

## 3 Discussion

This review has summarized the multifaceted pathophysiology of post-COVID, highlighting the complex interplay between dysregulated immune responses, a persistent viral reservoir, reactivation of latent viruses, vascular inflammation, neuronal and mitochondrial dysfunction, and microbiome dysbiosis. While much is known about acute SARS-CoV-2, significant gaps remain in understanding its long-term sequelae, particularly the differences between adult and pediatric presentations.

Evidence suggests that adult and pediatric post-COVID are overlapping but distinct entities. Adults experience a higher reported prevalence (9), and respiratory issues are more prominent, likely due to a lower incidence of severe acute lung damage in children. Children often mount a more robust innate immune response, potentially leading to faster viral clearance and a reduced risk of developing post-COVID. It is also crucial to consider the unique psychosocial context of children, as factors like school closures may contribute to symptom presentation and severity (235, 236). Finally, research on pediatric post-COVID is relatively limited, and more studies are needed to fully understand these distinctions in mechanisms, long-term consequences, and management strategies (9, 14).

A central challenge in understanding post-COVID is the high degree of patient heterogeneity. To move beyond symptom-based classification (237), researchers are using clustering methods to identify distinct clinical endotypes. These analyses, primarily in adults, have successfully identified patient sub-groups characterized by the involvement of specific organ systems (13, 55, 147, 238–245) (e.g., cardiovascular, neurocognitive, gastrointestinal) or by underlying biological signatures like sustained inflammatory responses, platelet degranulation, and metabolic disturbances (51, 78, 221, 239, 246–249). This work supports the view that PPCC is not a single disease but a syndrome comprising multiple conditions driven by interconnected disease mechanisms.

This complexity raises critical, unresolved questions about the role of each mechanistic axis.

A central, unresolved question highlighting this complexity is how acute COVID-19 leads to sustained immune dysregulation and whether this is a direct driver of post-COVID conditions or a consequence of other underlying factors. This complexity is evident within the adaptive immune system. For example, some patients show signs of chronic T-cell activation, with reduced naïve T-cell subsets and an increase in cytotoxic CD4^+^ and CD8^+^ T-cells. However, these findings are complicated by conflicting reports on the presence of exhausted T-cells and decreases in central and effector memory subsets, making the functional status of the T-cell compartment unclear. A key challenge is to determine whether this dysregulation is driven by persistent viral antigens, bystander activation from a dysbiotic microbiome or reactivated latent viruses, or a combination of these factors. Future studies investigating T-cell responses to reinfection could clarify the functional capacity of the immune system in these patients.

This variability extends to humoral immunity and antigen presentation. Studies have described antibody responses against S and N antigens as being equal to, reduced, or enhanced compared to recovered individuals, raising questions about how factors like a persistent viral reservoir or metabolic restrictions influence this response. Furthermore, while levels of conventional dendritic cells (cDCs) are reportedly decreased, there is an increase in antigen-presenting nonclassical and intermediate monocytes. This raises the critical question of whether these monocytes, or the inflammatory monocyte-derived dendritic cells (Mo-DCs) they can become, are the primary drivers of the T-cell activation observed in post-COVID, a crucial area that requires further research.

The hypothesis of viral persistence, while gaining traction as a potential contributing factor to post-COVID conditions, also raises several key questions. To what extent is viral persistence in various tissues explaining the breath of post-COVID symptoms? Is the persistent virus, if present, replication-competent, or non-replicating? Does persistent Spike antigen presence trigger cross-reactive immune responses, similar to those observed in some other post-infectious syndromes? During acute COVID-19, latent virus reactivation, particularly of herpesviruses like Epstein-Barr virus (EBV) and cytomegalovirus (CMV), has also been shown to exacerbate inflammation, potentially impairing type I interferon responses and skewing ^T-cell^ activation. Given that approximately 90% of the population latently carries EBV, why does reactivation not occur in all patients following COVID-19 infection (123, 124)? What is the direct role of reactivation and is there a potential treatment strategy targeting this phenomenon?

Gut and lung microbiome dysbiosis, triggered or exacerbated by SARS-CoV-2 infection, may contribute to post-COVID symptoms. The depletion of beneficial commensals and overgrowth of opportunistic pathogens can potentially lead to systemic inflammation, increased gut permeability, and immune dysregulation, potentially exacerbating neurocognitive and respiratory symptoms. Given the pivotal, protective role of butyrate-producing bacteria, the question of is there a (protective)? role for the tryptophan-kynurenine pathway in prevention of PPCC development? Another potential link between metabolic dysfunction and the gut microbiome concerns the reciprocal interactions between ROS and the gut epithelial barrier, and between the microbiota and mitochondrial respiratory chain function. Given the presence of an inflammatory environment and potentially lipoproteins in some post-COVID patients, could Mo-DCs play a role in maintaining an ‘inflammatory-bowel-disease-like’ state in certain individuals?

## 4 Conclusion

Ultimately, the multifactorial nature of post-COVID demands that future research prioritize identifying disease phenotypes based on underlying biology rather than symptoms alone. It is the complex interplay among the various mechanisms—where factors like viral persistence or microbiome dysbiosis may directly cause immune dysregulation and metabolic disturbances, which in turn drive downstream vascular and neuronal inflammation—that leads to the clinical syndrome. While all these factors are interconnected in a feedback loop, the evidence reviewed here points towards a dysregulated immune system as a dominant, though not exclusive, factor. For the many children and adolescents affected by PPCC, untangling this complexity through mechanism-based research is the only path toward improved diagnostics, targeted therapies, and the restoration of their future health.

## References

[1] Pierce CA Preston-Hurlburt P Dai Y Aschner CB Cheshenko N Galen B . Immune responses to SARS-CoV-2 infection in hospitalized pediatric and adult patients. Sci Trans Med. (2020) 12:1–10. doi: 10.1126/scitranslmed.abe8120, PMID: 32958614 PMC7658796

[2] Weisberg SP Connors TJ Zhu Y Baldwin MR Lin WH Wontakal S . Distinct antibody responses to SARS-CoV-2 in children and adults across the COVID-19 clinical spectrum. Nat Immunol. (2020) 22:25–31. doi: 10.1038/s41590-020-00826-9, PMID: 33154590 PMC8136619

[3] Davis HE McCorkell L Vogel JM Topol EJ . Long COVID: major findings, mechanisms and recommendations. Nat Rev Microbiol. (2023) 21:133–46. doi: 10.1038/s41579-022-00846-2, PMID: 36639608 PMC9839201

[4] Langeland N Cox RJ . Are low SARS-CoV-2 viral loads in infected children missed by RT-PCR testing? Lancet Reg Health – Eur. (2021) 5. doi: 10.1016/j.lanepe.2021.100138, PMID: 34075368 PMC8159172

[5] Zimmermann P Curtis N . Why is COVID-19 less severe in children? A review of the proposed mechanisms underlying the age-related difference in severity of SARS-CoV-2 infections. Arch Dis Childh. (2020), 1–11. doi: 10.1136/archdischild-2020-320338, PMID: 33262177

[6] Chen C Haupert SR Zimmermann L Shi X Fritsche LG Mukherjee B . Global prevalence of post COVID-19 condition or long COVID: A meta-analysis and systematic review. J Infect Dis. (2022) 226:1593–607. doi: 10.1093/INFDIS/JIAC136, PMID: 35429399 PMC9047189

[7] Su S Zhao Y Zeng N Liu X Zheng Y Sun J . Epidemiology, clinical presentation, pathophysiology, and management of long COVID: an update. Mol Psychiatry. (2023) 28:4056–69. doi: 10.1038/s41380-023-02171-3, PMID: 37491461

[8] Lap CR Brackel CLH Winkel AMAM Hashimoto S Haverkort M Noij LCE . Post-COVID-19 condition in children: epidemiological evidence stratified by acute disease severity. Pediatr Res. (2024), 1–9. doi: 10.1038/s41390-024-03597-3, PMID: 39333387

[9] Pazukhina E Andreeva M Spiridonova E Bobkova P Shikhaleva A El-Taravi Y . Prevalence and risk factors of post-COVID-19 condition in adults and children at 6 and 12 months after hospital discharge: a prospective, cohort study in Moscow (StopCOVID). BMC Med. (2022) 20:1–12. doi: 10.1186/s12916-022-02448-4, PMID: 35794549 PMC9257572

[10] Lopez-Leon S Wegman-Ostrosky T Ayuzo Del Valle NC Perelman C Sepulveda R Rebolledo PA . Long-COVID in children and adolescents: a systematic review and meta-analyses. Sci Rep. (2022) 12:9950. doi: 10.1038/s41598-022-13495-5, PMID: 35739136 PMC9226045

[11] Rao S Gross RS Mohandas S Stein CR Case A Dreyer B . Postacute sequelae of SARS-coV-2 in children. Pediatrics. (2024) 153. doi: 10.1542/peds.2023-062570, PMID: 38321938 PMC10904902

[12] Vahratian A Adjaye-Gbewonyo D Lin JMS Saydah S . Long COVID in children: United States, 2022. NCHS Data Br. (2023) 479):1–6. doi: 10.15620/cdc:132416 37756128

[13] Zheng YB Zeng N Yuan K Tian SS Yang YB Gao N . Prevalence and risk factor for long COVID in children and adolescents: A meta-analysis and systematic review. J Infect Public Health. (2023) 16:660–72. doi: 10.1016/j.jiph.2023.03.005, PMID: 36931142 PMC9990879

[14] Brodin P . Immune responses to SARS-CoV-2 infection and vaccination in children. Semin Immunol. (2023) 69:101794. doi: 10.1016/j.smim.2023.101794, PMID: 37536147 PMC10281229

[15] Stephenson T Pereira SMP Nugawela MD McOwat K Simmons R Chalder T . Long COVID—six months of prospective follow-up of changes in symptom profiles of non-hospitalised children and young people after SARS-CoV-2 testing: A national matched cohort study (The CLoCk) study. PloS One. (2023) 18:e0277704. doi: 10.1371/journal.pone.0277704, PMID: 36877677 PMC9987792

[16] Mantovani A Morrone MC Patrono C Santoro MG Schiaffino S Remuzzi G . Long Covid: where we stand and challenges ahead. Cell Death Diff. (2022) 29:1891–900. doi: 10.1038/s41418-022-01052-6, PMID: 36071155 PMC9449925

[17] (NICE) The National Institute for Health and Care Excellence . COVID-19 rapid guideline: managing the long-term effects of COVID-19. London (2020). 33555768

[18] Toepfner N Brinkmann F Augustin S Stojanov S Behrends U . Long COVID in pediatrics—epidemiology, diagnosis, and management. Eur J Pediatr. (2023). doi: 10.1007/s00431-023-05360-y, PMID: 38279014 PMC11001657

[19] World Health Organization . a clinical case definition for post covid-19 condition in children and adolescents by expert consensus. (2023), 1–99.

[20] Altmann DM Whettlock EM Liu S Arachchillage DJ Boyton RJ . The immunology of long COVID. Nat Rev Immunol. (2023) 23:618–34. doi: 10.1038/s41577-023-00904-7, PMID: 37433988

[21] Bosworth ML Shenhuy B Walker AS Nafilyan V Alwan NA O’Hara ME . Risk of new-onset long COVID following reinfection with severe acute respiratory syndrome coronavirus 2: A community-based cohort study. medRxiv. (2023) 10. doi: 10.1093/ofid/ofad493, PMID: 37953820 PMC10633780

[22] Bowe B Xie Y Al-Aly Z . Acute and postacute sequelae associated with SARS-CoV-2 reinfection. Nat Med. (2022) 28:2398–405. doi: 10.1038/s41591-022-02051-3, PMID: 36357676 PMC9671810

[23] De Bruijn S Van Hoek AJ Mutubuki EN Knoop H Slootweg J Tulen AD . Lower prevalence of Post-Covid-19 Condition following Omicron SARS-CoV-2 infection. medRxiv. (2023), 1–20. doi: 10.1101/2023.04.05.23288157, PMID: 38617937 PMC11015416

[24] Sudre CH Murray B Varsavsky T Graham MS Penfold RS Bowyer RC . Attributes and predictors of long COVID. Nat Med. (2021) 27:626–31. doi: 10.1038/s41591-021-01292-y, PMID: 33692530 PMC7611399

[25] Behnood SA Shafran R Bennett SD Zhang AXD O’Mahoney LL Stephenson TJ . Persistent symptoms following SARS-CoV-2 infection amongst children and young people: A meta-analysis of controlled and uncontrolled studies. J Infect. (2022) 84:158–70. doi: 10.1016/j.jinf.2021.11.011, PMID: 34813820 PMC8604800

[26] Morrow AK Villatoro C Kokorelis C Rowe PC Malone LA . Orthostatic intolerance in children with long COVID utilizing a 10-minute passive standing test. Clin Pediatr (Phila). (2024), 00099228241272053. doi: 10.1177/00099228241272053, PMID: 39123312 PMC12444382

[27] Fedorowski A Fanciulli A Raj SR Sheldon R Shibao CA Sutton R . Cardiovascular autonomic dysfunction in post-COVID-19 syndrome: a major health-care burden. Nat Rev Cardiol. (2024). doi: 10.1038/s41569-023-00962-3, PMID: 38163814

[28] Raman B Bluemke DA Lüscher TF Neubauer S . Long COVID: post-acute sequelae of COVID-19 with a cardiovascular focus. Eur Heart J. (2022) 43:1157–72. doi: 10.1093/EURHEARTJ/EHAC031, PMID: 35176758 PMC8903393

[29] Camporesi A Morello R Rocca AL Zampino G Vezzulli F Munblit D . Characteristics and predictors of Long Covid in children: a 3-year prospective cohort study. eClinicalMedicine. (2024) 76. doi: 10.1016/j.eclinm.2024.102815, PMID: 39296584 PMC11408803

[30] Iwasaki A Putrino D . Why we need a deeper understanding of the pathophysiology of long COVID. Lancet Infect Dis. (2023) 23:393–5. doi: 10.1016/S1473-3099(23)00053-1, PMID: 36967698 PMC9928485

[31] Subramanian A Nirantharakumar K Hughes S Myles P Williams T Gokhale KM . Symptoms and risk factors for long COVID in non-hospitalized adults. Nat Med. (2022) 28:1706–14. doi: 10.1038/s41591-022-01909-w, PMID: 35879616 PMC9388369

[32] Robertson MM Qasmieh SA Kulkarni SG Teasdale CA Jones HE McNairy M . The epidemiology of long coronavirus disease in US adults. Clin Infect Dis. (2023) 76:1636–45. doi: 10.1093/CID/CIAC961, PMID: 36542514

[33] Sherif ZA Gomez CR Connors TJ Henrich TJ Reeves WB . Pathogenic mechanisms of post-acute sequelae of SARS-CoV-2 infection (PASC). eLife. (2023) 12:1–31. doi: 10.7554/elife.86002, PMID: 36947108 PMC10032659

[34] Al-aly BZ Topol E . Solving the puzzle of long covid. (2024) 383:830–2. doi: 10.1126/science.adl0867, PMID: 38386747

[35] Li J Zhou Y Ma J Zhang Q Shao J Liang S . The long-term health outcomes, pathophysiological mechanisms and multidisciplinary management of long COVID. Signal Transd Targ Ther. (2023) 8:1–19. doi: 10.1038/s41392-023-01640-z, PMID: 37907497 PMC10618229

[36] Guo L Appelman B Mooij-Kalverda K Houtkooper RH van Weeghel M Vaz FM . Prolonged indoleamine 2,3-dioxygenase-2 activity and associated cellular stress in post-acute sequelae of SARS-CoV-2 infection. eBioMedicine. (2023) 94. doi: 10.1016/j.ebiom.2023.104729, PMID: 37506544 PMC10406961

[37] Topol E . Long covid: mitochondria, the big miss, and hope. Available online at: https://erictopol.substack.com/p/long-covid-mitochondria-the-big-miss (Accessed August 13, 2023).

[38] Yin K Peluso MJ Luo X Thomas R Shin MG Neidleman J . Long COVID manifests with T cell dysregulation, inflammation and an uncoordinated adaptive immune response to SARS-CoV-2. Nat Immunol. (2024). doi: 10.1038/s41590-023-01724-6, PMID: 38212464 PMC10834368

[39] Peppercorn K Edgar CD Kleffmann T Tate WP . A pilot study on the immune cell proteome of long COVID patients shows changes to physiological pathways similar to those in myalgic encephalomyelitis/chronic fatigue syndrome. Sci Rep. (2023) 13:1–14. doi: 10.1038/s41598-023-49402-9, PMID: 38086949 PMC10716514

[40] Merad M Blish CA Sallusto F Iwasaki A . The immunology and immunopathology of COVID-19. Science. (2022) 375:1122–7. doi: 10.1126/science.abm8108, PMID: 35271343 PMC12828912

[41] Qiao W Lau HE Xie H Poon VKM Chan CCS Chu H . SARS-CoV-2 infection induces inflammatory bone loss in golden Syrian hamsters. Nat Commun. (2022) 13:2539. doi: 10.1038/s41467-022-30195-w, PMID: 35534483 PMC9085785

[42] Costagliola G Spada E Consolini R . Age-related differences in the immune response could contribute to determine the spectrum of severity of COVID-19. Immun Inflammation Dis. (2021) 9:331–9. doi: 10.1002/iid3.404, PMID: 33566457 PMC8014746

[43] Ng PC Lam CWK Li AM Wong CK Cheng FWT Leung TF . Inflammatory cytokine profile in children with severe acute respiratory syndrome. Pediatrics. (2004) 113:e7–14. doi: 10.1542/peds.113.1.e7, PMID: 14702488

[44] Loske J Röhmel J Lukassen S Stricker S Magalhães VG Liebig J . Pre-activated antiviral innate immunity in the upper airways controls early SARS-CoV-2 infection in children. Nat Biotechnol. (2022) 40:319–24. doi: 10.1038/s41587-021-01037-9, PMID: 34408314

[45] Howard-Jones AR Burgner DP Crawford NW Goeman E Gray PE Hsu P . COVID-19 in children. II: Pathogenesis, disease spectrum and management. J Paediatr Child Health. (2022) 58:46–53. doi: 10.1111/JPC.15811, PMID: 34694037 PMC8662268

[46] Phetsouphanh C Darley DR Wilson DB Howe A Munier CML Patel SK . Immunological dysfunction persists for 8 months following initial mild-to-moderate SARS-CoV-2 infection. Nat Immunol. (2022) 23:210–6. doi: 10.1038/s41590-021-01113-x, PMID: 35027728

[47] Colonna M Trinchieri G Liu YJ . Plasmacytoid dendritic cells in immunity. Nat Immunol. (2004) 5:1219–26. doi: 10.1038/ni1141, PMID: 15549123

[48] Merad M Sathe P Helft J Miller J Mortha A . The dendritic cell lineage: ontogeny and function of dendritic cells and their subsets in the steady state and the inflamed setting. Annu Rev Immunol. (2013) 31:563–604. doi: 10.1146/ANNUREV-IMMUNOL-020711-074950, PMID: 23516985 PMC3853342

[49] Sasaki I Kato T Hemmi H Fukuda-Ohta Y Wakaki-Nishiyama N Yamamoto A . Conventional type 1 dendritic cells in intestinal immune homeostasis. Front Immunol. (2022) 13:857954/BIBTEX. doi: 10.3389/fimmu.2022.857954, PMID: 35693801 PMC9184449

[50] Yoshida M Worlock KB Huang N Lindeboom RGH Butler CR Kumasaka N . Local and systemic responses to SARS-CoV-2 infection in children and adults. Nature. (2021) 602:321–7. doi: 10.1038/s41586-021-04345-x, PMID: 34937051 PMC8828466

[51] Klein J Wood J Jaycox JR Dhodapkar RM Lu P Gehlhausen JR . Distinguishing features of long COVID identified through immune profiling. Nature. (2023) 623:139–48. doi: 10.1038/s41586-023-06651-y, PMID: 37748514 PMC10620090

[52] Hopkins FR Govender M Svanberg C Nordgren J Waller H Nilsdotter-Augustinsson Å . Major alterations to monocyte and dendritic cell subsets lasting more than 6 months after hospitalization for COVID-19. Front Immunol. (2023) 13:1082912/BIBTEX. doi: 10.3389/fimmu.2022.1082912, PMID: 36685582 PMC9846644

[53] Venet M Ribeiro MS Décembre E Bellomo A Joshi G Nuovo C . Severe COVID-19 patients have impaired plasmacytoid dendritic cell-mediated control of SARS-CoV-2. Nat Commun. (2023) 14:694. doi: 10.1038/s41467-023-36140-9, PMID: 36755036 PMC9907212

[54] Thompson RC Simons NW Wilkins L Cheng E Del Valle DM Hoffman GE . Molecular states during acute COVID-19 reveal distinct etiologies of long-term sequelae. Nat Med. (2023) 29:236–46. doi: 10.1038/s41591-022-02107-4, PMID: 36482101 PMC9873574

[55] Liew F Openshaw P Efstathiou C Fontanella S Richardson M Saunders R . Large scale phenotyping of long COVID inflammation reveals mechanistic subtypes of disease after COVID-19 hospitalisation. Res Sq. (2023). doi: 10.21203/rs.3.rs-3427282/v1, PMID: 41884470

[56] Rodriguez L Tan Z Lakshmikanth T Wang J Brodin P . Restrained memory CD8 + T cell responses favors viral persistence and elevated IgG responses in patients with severe Long COVID. MedRxiv. (2024). doi: 10.1101/2024.02.11.24302636, PMID: 41887800

[57] Narasimhan PB Marcovecchio P Hamers AAJ Hedrick CC . Nonclassical monocytes in health and disease. Annu Rev Immunol. (2019) 37:439–56. doi: 10.1146/annurev-immunol-042617-053119, PMID: 31026415

[58] Berentschot JC Drexhage HA Aynekulu Mersha DG Wijkhuijs AJM GeurtsvanKessel CH Koopmans MPG . Immunological profiling in long COVID: overall low grade inflammation and T-lymphocyte senescence and increased monocyte activation correlating with increasing fatigue severity. Front Immunol. (2023) 14:1254899/BIBTEX. doi: 10.3389/fimmu.2023.1254899, PMID: 37881427 PMC10597688

[59] Kapellos TS Bonaguro L Gemünd I Reusch N Saglam A Hinkley ER . Human monocyte subsets and phenotypes in major chronic inflammatory diseases. Front Immunol. (2019) 10:2035. doi: 10.3389/fimmu.2019.02035, PMID: 31543877 PMC6728754

[60] Witkowski M Tizian C Ferreira-Gomes M Niemeyer D Jones TC Heinrich F . Untimely TGFβ responses in COVID-19 limit antiviral functions of NK cells. Nature. (2021) 600:295–301. doi: 10.1038/s41586-021-04142-6, PMID: 34695836

[61] Galán M Vigón L Fuertes D Murciano-Antón MA Casado-Fernández G Domínguez-Mateos S . Persistent overactive cytotoxic immune response in a spanish cohort of individuals with long-COVID: identification of diagnostic biomarkers. Front Immunol. (2022) 13:848886. doi: 10.3389/fimmu.2022.848886, PMID: 35401523 PMC8990790

[62] Seliger B Jasinski-Bergner S Quandt D Stoehr C Bukur J Wach S . HLA-E expression and its clinical relevance in human renal cell carcinoma. Oncotarget. (2016) 7:67360–72. doi: 10.18632/oncotarget.11744, PMID: 27589686 PMC5341881

[63] Monaco EL Tremante E Cerboni C Melucci E Sibilio L Zingoni A . Human leukocyte antigen E contributes to protect tumor cells from lysis by natural killer cells. Neoplasia. (2011) 13:822–IN14. doi: 10.1593/neo.101684, PMID: 21969815 PMC3182274

[64] Huot N Planchais C Rosenbaum P Contreras V Jacquelin B Petitdemange C . SARS-CoV-2 viral persistence in lung alveolar macrophages is controlled by IFN-γ and NK cells. Nat Immunol. (2023) 24:2068–79. doi: 10.1038/s41590-023-01661-4, PMID: 37919524 PMC10681903

[65] Cervia-Hasler C Brüningk SC Hoch T Fan B Muzio G Thompson RC . Persistent complement dysregulation with signs of thromboinflammation in active Long Covid. Science. (2024) 383. doi: 10.1126/science.adg7942, PMID: 38236961

[66] Baillie K Davies HE Keat SBK Ladell K Miners KL Jones SA . Complement dysregulation is a prevalent and therapeutically amenable feature of long COVID. Med. (2024) 5:239–253.e5. doi: 10.1016/j.medj.2024.01.011, PMID: 38359836

[67] Di Sante G Buonsenso D De Rose C Tredicine M Palucci I De Maio F . Immunopathology of SARS-coV-2 infection: A focus on T regulatory and B cell responses in children compared with adults. Children. (2022) 9. doi: 10.3390/children9050681, PMID: 35626859 PMC9139466

[68] Chen B Julg B Mohandas S Bradfute SB . Viral persistence, reactivation, and mechanisms of long COVID. eLife. (2023) 12:1–15. doi: 10.7554/eLife.86015, PMID: 37140960 PMC10159620

[69] Peluso MJ Deitchman AN Torres L Iyer NS Munter SE Nixon CC . Long-term SARS-CoV-2-specific immune and inflammatory responses in individuals recovering from COVID-19 with and without post-acute symptoms. Cell Rep. (2021) 36. doi: 10.1016/j.celrep.2021.109518, PMID: 34358460 PMC8342976

[70] Di Sante G Buonsenso D De Rose C Tredicine M Palucci I De Maio F . Immunopathology of SARS-coV-2 infection: A focus on T regulatory and B cell responses in children compared with adults. Children. (2022) 9. doi: 10.3390/children9050681, PMID: 35626859 PMC9139466

[71] Aiello A Farzaneh F Candore G Caruso C Davinelli S Gambino CM . Immunosenescence and its hallmarks: how to oppose aging strategically? A review of potential options for therapeutic intervention. Front Immunol. (2019) 10:2247. doi: 10.3389/FIMMU.2019.02247, PMID: 31608061 PMC6773825

[72] Glynne P Tahmasebi N Gant V Gupta R . Long COVID following mild SARS-coV-2 infection: characteristic T cell alterations and response to antihistamines. J Invest Med. (2022) 70:61–7. doi: 10.1136/jim-2021-002051, PMID: 34611034 PMC8494538

[73] Nalbandian A Sehgal K Gupta A Madhavan MV McGroder C Stevens JS . Post-acute COVID-19 syndrome. Nat Med. (2021) 27:601–15. doi: 10.1038/s41591-021-01283-z, PMID: 33753937 PMC8893149

[74] Talla A Vasaikar SV Lemos MP Moodie Z Lee Pebworth M-P Henderson KE . Longitudinal immune dynamics of mild COVID-19 define signatures of recovery and persistence. bioRxiv. (2021), 442666. doi: 10.1101/2021.05.26.442666, PMID: 34075380 PMC8168393

[75] Visvabharathy L Hanson BA Orban ZS Lim PH Palacio NM Jimenez M . T cell responses to SARS-CoV-2 in people with and without neurologic symptoms of long COVID. medRxiv. (2022). doi: 10.1101/2021.08.08.21261763, PMID: 34401886 PMC8366804

[76] Krishna BA Lim EY Mactavous L Jackson S Lyons PA Bradley JR . Spontaneous, persistent T-cell dependent IFN- γ release in patients who progress to Long COVID. Sci Adv. (2024) 10. doi: 10.1126/sciadv.adi9379, PMID: 38381822 PMC10881041

[77] LaVergne SM Dutt TS McFann K Baxter BA Webb TL Berry K . Persistent CD8+ T cell proliferation and activation in COVID-19 adult survivors with post-acute sequelae: a longitudinal, observational cohort study of persistent symptoms and T cell markers. Front Immunol. (2024) 14:1303971. doi: 10.3389/fimmu.2023.1303971, PMID: 38327763 PMC10848319

[78] Su Y Yuan D Chen DG Ng RH Wang K Choi J . Multiple early factors anticipate post-acute COVID-19 sequelae. Cell. (2022) 185:881–895.e20. doi: 10.1016/j.cell.2022.01.014, PMID: 35216672 PMC8786632

[79] Wiech M Chroscicki P Swatler J Stepnik D De Biasi S Hampel M . Remodeling of T cell dynamics during long COVID is dependent on severity of SARS-coV-2 infection. Front Immunol. (2022) 13:886431. doi: 10.3389/fimmu.2022.886431, PMID: 35757700 PMC9226563

[80] Visvabharathy L Hanson BA Orban ZS Lim PH Palacio NM Jimenez M . Neuro-PASC is characterized by enhanced CD4+ and diminished CD8+ T cell responses to SARS-CoV-2 Nucleocapsid protein. Front Immunol. (2023) 14:1155770/BIBTEX. doi: 10.3389/fimmu.2023.1155770, PMID: 37313412 PMC10258318

[81] Crotty S . Follicular helper CD4 T cells (TFH). Annu Rev Immunol. (2011) 29:621–63. doi: 10.1146/annurev-immunol-031210-101400, PMID: 21314428

[82] Mohandas S Jagannathan P Henrich TJ Sherif ZA Bime C Quinlan E . Immune mechanisms underlying COVID-19 pathology and post-acute sequelae of SARS-CoV-2 infection (PASC). eLife. (2023) 12:1–19. doi: 10.7554/eLife.86014, PMID: 37233729 PMC10219649

[83] Augustin M Schommers P Stecher M Dewald F Gieselmann L Gruell H . Post-COVID syndrome in non-hospitalised patients with COVID-19: a longitudinal prospective cohort study. Lancet Reg Health - Eur. (2021) 6. doi: 10.1016/j.lanepe.2021.100122, PMID: 34027514 PMC8129613

[84] García-Abellán J Padilla S Fernández-González M García JA Agulló V Andreo M . Antibody response to SARS-coV-2 is associated with long-term clinical outcome in patients with COVID-19: a longitudinal study. J Clin Immunol. (2021) 41:1490–501. doi: 10.1007/s10875-021-01083-7, PMID: 34273064 PMC8285689

[85] Jia X Cao S Lee AS Manohar M Sindher SB Ahuja N . Anti-nucleocapsid antibody levels and pulmonary comorbid conditions are linked to post–COVID-19 syndrome. JCI Insight. (2022) 7. doi: 10.1172/JCI.INSIGHT.156713, PMID: 35801588 PMC9310538

[86] Vojdani A Vojdani E Saidara E Maes M . Persistent SARS-coV-2 infection, EBV, HHV-6 and other factors may contribute to inflammation and autoimmunity in long COVID. Viruses. (2023) 15. doi: 10.3390/v15020400, PMID: 36851614 PMC9967513

[87] Vojdani A Kharrazian D . Potential antigenic cross-reactivity between SARS-CoV-2 and human tissue with a possible link to an increase in autoimmune diseases. Clin Immunol. (2020) 217:108480. doi: 10.1016/J.CLIM.2020.108480, PMID: 32461193 PMC7246018

[88] Bodansky A Mettelman RC Sabatino JJ Vazquez SE Chou J Novak T . Molecular mimicry in multisystem inflammatory syndrome in children. Nature. (2024) 632:622–9. doi: 10.1038/s41586-024-07722-4, PMID: 39112696 PMC11324515

[89] ChenHJ AppelmanB WillemenH BosA PradoJ GeyerCE . Transfer of IgG from Long COVID patients induces symptomology in mice. bioRxiv. (2024). doi: 10.1101/2024.05.30.596590, PMID: 41881023

[90] Rojas M Rodríguez Y Acosta-Ampudia Y Monsalve DM Zhu C Li QZ . Autoimmunity is a hallmark of post-COVID syndrome. J Trans Med. (2022) 20:1–5. doi: 10.1186/s12967-022-03328-4, PMID: 35296346 PMC8924736

[91] Son K Jamil R Chowdhury A Mukherjee M Venegas C Miyasaki K . Circulating anti-nuclear autoantibodies in COVID-19 survivors predict long COVID symptoms. Eur Respir J. (2023) 61. doi: 10.1183/13993003.00970-2022, PMID: 36137590 PMC9515477

[92] Wallukat G Hohberger B Wenzel K Fürst J Schulze-Rothe S Wallukat A . Functional autoantibodies against G-protein coupled receptors in patients with persistent Long-COVID-19 symptoms. J Trans Autoimmun. (2021) 4:100100. doi: 10.1016/J.JTAUTO.2021.100100, PMID: 33880442 PMC8049853

[93] Schultheiß C Willscher E Paschold L Gottschick C Klee B Henkes SS . The IL-1β, IL-6, and TNF cytokine triad is associated with post-acute sequelae of COVID-19. Cell Rep Med. (2022) 3. doi: 10.1016/j.xcrm.2022.100663, PMID: 35732153 PMC9214726

[94] Deen GF Broutet N Xu W Knust B Sesay FR McDonald SLR . Ebola RNA persistence in semen of ebola virus disease survivors — Final report. New Engl J Med. (2017) 377:1428–37. doi: 10.1056/nejmoa1511410, PMID: 26465681 PMC5798881

[95] Dokubo EK Wendland A Mate SE Ladner JT Hamblion EL Raftery P . Persistence of Ebola virus after the end of widespread transmission in Liberia: an outbreak report. Lancet Infect Dis. (2018) 18:1015–24. doi: 10.1016/S1473-3099(18)30417-1, PMID: 30049622

[96] Keita AK Vidal N Toure A Kalifa Diallo MS N’Fally M Baize S . A 40-month follow-up of ebola virus disease survivors in Guinea (PostEbogui) reveals long-term detection of ebola viral ribonucleic acid in semen and breast milk. Open Forum Infect Dis. (2019) 6. doi: 10.1093/OFID/OFZ482, PMID: 32128327 PMC7047953

[97] Zuo W He D Liang C Du S Hua Z Nie Q . The persistence of SARS-CoV-2 in tissues and its association with long COVID symptoms: a cross-sectional cohort study in China. Lancet Infect Dis. (2024) 24:845–55. doi: 10.1016/S1473-3099(24)00171-3, PMID: 38663423

[98] Herrera-Valdés R Almaguer-López M López-Marín L Bacallao-Méndez R Pérez-Oliva JF Guerra-Bustillo G . COVID-19 and the kidneys: risk, damage and sequelae. MEDICC Rev. (2020) 22:87–8. doi: 10.37757/MR2020.V22.N4.10, PMID: 33295327

[99] Su H Yang M Wan C Yi LX Tang F Zhu HY . Renal histopathological analysis of 26 postmortem findings of patients with COVID-19 in China. Kidney Int. (2020) 98:219–27. doi: 10.1016/J.KINT.2020.04.003, PMID: 32327202 PMC7194105

[100] Cevik M Tate M Lloyd O Maraolo AE Schafers J Ho A . SARS-CoV-2, SARS-CoV, and MERS-CoV viral load dynamics, duration of viral shedding, and infectiousness: a systematic review and meta-analysis. Lancet Microbe. (2021) 2:e13–22. doi: 10.1016/s2666-5247(20)30172-5, PMID: 33521734 PMC7837230

[101] Gamage AM Tan KS Chan WOY Lew ZZR Liu J Tan CW . Human nasal epithelial cells sustain persistent SARS-coV-2 infection *in vitro*, despite eliciting a prolonged antiviral response. mBio. (2022) 13. doi: 10.1128/mbio.03436-21, PMID: 35038898 PMC8764519

[102] Lee S Yoon GY Myoung J Kim SJ Ahn DG . Robust and persistent SARS-CoV-2 infection in the human intestinal brush border expressing cells. Emerg Microbes Infect. (2020) 9:2169–79. doi: 10.1080/22221751.2020.1827985, PMID: 32969768 PMC7580600

[103] Ghafari M Hall M Golubchik T Ayoubkhani D House T MacIntyre-Cockett G . Prevalence of persistent SARS-CoV-2 in a large community surveillance study. Nature. (2024) 626:1094–101. doi: 10.1038/s41586-024-07029-4, PMID: 38383783 PMC10901734

[104] Proal AD VanElzakker MB Aleman S Bach K Boribong BP Buggert M . SARS-CoV-2 reservoir in post-acute sequelae of COVID-19 (PASC). Nat Immunol. (2023) 24:1616–27. doi: 10.1038/s41590-023-01601-2, PMID: 37667052

[105] Sattentau Q . Avoiding the void: cell-to-cell spread of human viruses. Nat Rev Microbiol. (2008) 6:815–26. doi: 10.1038/nrmicro1972, PMID: 18923409

[106] Zeng C Evans JP King T Zheng YM Oltz EM Whelan SPJ . SARS-CoV-2 spreads through cell-to-cell transmission. Proc Natl Acad Sci Utd States America. (2022) 119:e2111400119. doi: 10.1073/pnas.2111400119, PMID: 34937699 PMC8740724

[107] Turner S Khan MA Putrino D Woodcock A Kell DB Pretorius E . Long COVID: pathophysiological factors and abnormalities of coagulation. Trends Endocrinol Metab. (2023) 34:321–44. doi: 10.1016/j.tem.2023.03.002, PMID: 37080828 PMC10113134

[108] Borowiec BM Angelova Volponi A Mozdziak P Kempisty B Dyszkiewicz-Konwińska M . Small extracellular vesicles and COVID19—Using the “Trojan horse” to tackle the giant. Cells. (2021) 10:12. doi: 10.3390/cells10123383, PMID: 34943891 PMC8699232

[109] Chiappini E Licari A Motisi MA Manti S Marseglia GL Galli L . Gastrointestinal involvement in children with SARS-COV-2 infection: An overview for the pediatrician. Pediatr Allergy Immunol. (2020) 31:92–5. doi: 10.1111/pai.13373, PMID: 33236437 PMC7753808

[110] Natarajan A Zlitni S Brooks EF Vance SE Dahlen A Hedlin H . Gastrointestinal symptoms and fecal shedding of SARS-CoV-2 RNA suggest prolonged gastrointestinal infection. Med. (2022) 3:371–387.e9. doi: 10.1016/j.medj.2022.04.001, PMID: 35434682 PMC9005383

[111] Du W Yu J Liu X Chen H Lin L Li Q . Persistence of SARS-CoV-2 virus RNA in feces: A case series of children. J Infect Public Health. (2020) 13:926–31. doi: 10.1016/j.jiph.2020.05.025, PMID: 32546439 PMC7275988

[112] Xu CLH Raval M Schnall JA Kwong JC Holmes NE . Duration of respiratory and gastrointestinal viral shedding in children with SARS-coV-2: A systematic review and synthesis of data. Pediatr Infect Dis J. (2020) 39:e249. doi: 10.1097/INF.0000000000002814, PMID: 32618932

[113] Hu F Chen F Ou Z Fan Q Tan X Wang Y . A compromised specific humoral immune response against the SARS-CoV-2 receptor-binding domain is related to viral persistence and periodic shedding in the gastrointestinal tract. Cell Mol Immunol. (2020) 17:1119–25. doi: 10.1038/s41423-020-00550-2, PMID: 33037400 PMC7546387

[114] Gaebler C Wang Z Lorenzi JCC Muecksch F Finkin S Tokuyama M . Evolution of antibody immunity to SARS-CoV-2. Nature. (2021) 591:639–44. doi: 10.1038/s41586-021-03207-w, PMID: 33461210 PMC8221082

[115] Yonker LM Gilboa T Ogata AF Senussi Y Lazarovits R Boribong BP . Multisystem inflammatory syndrome in children is driven by zonulin-dependent loss of gut mucosal barrier. J Clin Invest. (2021) 131. doi: 10.1172/JCI149633, PMID: 34032635 PMC8279585

[116] Brodin P . SARS-CoV-2 infections in children: Understanding diverse outcomes. Immunity. (2022) 55:201–9. doi: 10.1016/j.immuni.2022.01.014, PMID: 35093190 PMC8769938

[117] Buonsenso D Tantisira KG . Long COVID and SARS-CoV-2 persistence: new answers, more questions. Lancet Infect Dis. (2024) 24:796–8. doi: 10.1016/s1473-3099(24)00216-0, PMID: 38663424

[118] Peluso MJ Lu S Tang AF Durstenfeld MS Ho HE Goldberg SA . Markers of immune activation and inflammation in individuals with postacute sequelae of severe acute respiratory syndrome coronavirus 2 infection. J Infect Dis. (2021) 224:1839–48. doi: 10.1093/INFDIS/JIAB490, PMID: 34677601 PMC8643408

[119] Littlefield KM Watson RO Schneider JM Neff CP Yamada E Zhang M . SARS-CoV-2-specific T cells associate with inflammation and reduced lung function in pulmonary post-acute sequalae of SARS-CoV-2. PloS Pathog. (2022) 18. doi: 10.1371/journal.ppat.1010359, PMID: 35617421 PMC9176759

[120] Simonnet A Engelmann I Moreau AS Garcia B Six S El Kalioubie A . High incidence of Epstein–Barr virus, cytomegalovirus, and human-herpes virus-6 reactivations in critically ill patients with COVID-19. Infect Dis Now. (2021) 51:296–9. doi: 10.1016/j.idnow.2021.01.005, PMID: 33495765 PMC7816954

[121] Zubchenko S Kril I Nadizhko O Matsyura O Chopyak V . Herpesvirus infections and post-COVID-19 manifestations: a pilot observational study. Rheumatol Int. (2022) 42:1523–30. doi: 10.1007/s00296-022-05146-9, PMID: 35650445 PMC9159383

[122] Peluso MJ Deveau TM Munter SE Ryder D Buck A Beck-Engeser G . Chronic viral coinfections differentially affect the likelihood of developing long COVID. J Clin Invest. (2023) 133. doi: 10.1172/JCI163669, PMID: 36454631 PMC9888380

[123] Cohen JI . Epstein-Barr virus infection. New Engl J Med. (2000) 343:481–92. doi: 10.1056/NEJM200008173430707, PMID: 10944566

[124] Gold JE Okyay RA Licht WE Hurley DJ . Investigation of long COVID prevalence and its relationship to epstein-barr virus reactivation. Pathogens. (2021) 10:763. doi: 10.3390/PATHOGENS10060763, PMID: 34204243 PMC8233978

[125] Verma D Church TM Swaminathan S . Epstein-barr virus lytic replication induces ACE2 expression and enhances SARS-coV-2 pseudotyped virus entry in epithelial cells. J Virol. (2021) 95. doi: 10.1128/jvi.00192-21, PMID: 33853968 PMC8316011

[126] Hashimoto K . Detrimental effects of COVID-19 in the brain and therapeutic options for long COVID: The role of Epstein–Barr virus and the gut–brain axis. Mol Psychiatry. (2023), 1–9. doi: 10.1038/s41380-023-02161-5, PMID: 37402856 PMC11041741

[127] Hu Z Li S Song X . Cytokine storm with rapidly elevated interleukin-6 indicates sudden death in patients with critical COVID-19. Cytokine Growth F Rev. (2021) 58:30–1. doi: 10.1016/J.CYTOGFR.2020.08.001, PMID: 32873506 PMC7445135

[128] Chaudhary R Kreutz RP Bliden KP Tantry US Gurbel PA . Personalizing antithrombotic therapy in COVID-19: role of thromboelastography and thromboelastometry. Thromb Haemost. (2020) 120:1594–6. doi: 10.1055/s-0040-1714217, PMID: 32679595

[129] Oikonomou E Souvaliotis N Lampsas S Siasos G Poulakou G Theofilis P . Endothelial dysfunction in acute and long standing COVID−19: A prospective cohort study. Vasc Pharmacol. (2022) 144:106975. doi: 10.1016/J.VPH.2022.106975, PMID: 35248780 PMC8893931

[130] Pavoni V Gianesello L Pazzi M Stera C Meconi T Frigieri FC . Evaluation of coagulation function by rotation thromboelastometry in critically ill patients with severe COVID-19 pneumonia. J Thromb Thrombol. (2020) 50:281–6. doi: 10.1007/s11239-020-02130-7, PMID: 32394236 PMC7211560

[131] Buonsenso D Gennaro LD Rose CD Morello R D’Ilario F Zampino G . Long-term outcomes of pediatric infections: from traditional infectious diseases to long covid. Future Microbiol. (2022). doi: 10.2217/fmb-2022-0031, PMID: 35264003 PMC8910780

[132] Di Gennaro L Valentini P Sorrentino S Ferretti MA De Candia E Basso M . Extended coagulation profile of children with Long Covid: a prospective study. Sci Rep. (2022) 12:1–10. doi: 10.1038/s41598-022-23168-y, PMID: 36319840 PMC9626616

[133] Kell DB Laubscher GJ Pretorius E . A central role for amyloid fibrin microclots in long COVID/PASC: origins and therapeutic implications. Biochem J. (2022) 479:537–59. doi: 10.1042/BCJ20220016, PMID: 35195253 PMC8883497

[134] Lee MH Perl DP Steiner J Pasternack N Li W Maric D . Neurovascular injury with complement activation and inflammation in COVID-19. Brain. (2022) 145:2555–68. doi: 10.1093/BRAIN/AWAC151, PMID: 35788639 PMC9278212

[135] Huppert LA Matthay MA Ware LB . Pathogenesis of acute respiratory distress syndrome. Semin Respir Crit Care Med. (2019) 40:31–9. doi: 10.1055/s-0039-1683996, PMID: 31060086 PMC7060969

[136] Kubánková M Hohberger B Hoffmanns J Fürst J Herrmann M Guck J . Physical phenotype of blood cells is altered in COVID-19. Biophys J. (2021) 120:2838–47. doi: 10.1016/j.bpj.2021.05.025, PMID: 34087216 PMC8169220

[137] Osiaevi I Schulze A Evers G Harmening K Vink H Kümpers P . Persistent capillary rarefication in long COVID syndrome. Angiogenesis. (2023) 26:53–61. doi: 10.1007/s10456-022-09850-9, PMID: 35951203 PMC9366128

[138] Szewczykowski C Mardin C Lucio M Wallukat G Hoffmanns J Schröder T . Long COVID: association of functional autoantibodies against G-protein-coupled receptors with an impaired retinal microcirculation. Int J Mol Sci. (2022) 23:7209. doi: 10.3390/ijms23137209, PMID: 35806214 PMC9266742

[139] Haffke M Freitag H Rudolf G Seifert M Doehner W Scherbakov N . Endothelial dysfunction and altered endothelial biomarkers in patients with post-COVID-19 syndrome and chronic fatigue syndrome (ME/CFS). J Trans Med. (2022) 20:1–11. doi: 10.1186/s12967-022-03346-2, PMID: 35317812 PMC8938726

[140] Calabretta E Moraleda JM Iacobelli M Jara R Vlodavsky I O’Gorman P . COVID-19-induced endotheliitis: emerging evidence and possible therapeutic strategies. Br J Haematol. (2021) 193:43–51. doi: 10.1111/bjh.17240, PMID: 33538335 PMC8014053

[141] Pretorius E Vlok M Venter C Bezuidenhout JA Laubscher GJ Steenkamp J . Persistent clotting protein pathology in Long COVID/Post-Acute Sequelae of COVID-19 (PASC) is accompanied by increased levels of antiplasmin. Cardiovasc Diabetol. (2021) 20:1–18. doi: 10.1186/s12933-021-01359-7, PMID: 34425843 PMC8381139

[142] Kruger A Vlok M Turner S Venter C Laubscher GJ Kell DB . Proteomics of fibrin amyloid microclots in long COVID/post-acute sequelae of COVID-19 (PASC) shows many entrapped pro-inflammatory molecules that may also contribute to a failed fibrinolytic system. Cardiovasc Diabetol. (2022) 21:1–23. doi: 10.1186/s12933-022-01623-4, PMID: 36131342 PMC9491257

[143] Buonsenso D Cotugno N Amodio D Pascucci GR Di Sante G Pighi C . Distinct pro-inflammatory/pro-angiogenetic signatures distinguish children with Long COVID from controls. Pediatr Res. (2025), 1–8. doi: 10.1038/s41390-025-03837-0, PMID: 39849114

[144] Pober JS Sessa WC . Evolving functions of endothelial cells in inflammation. Nat Rev Immunol. (2007) 7:803–15. doi: 10.1038/nri2171, PMID: 17893694

[145] Teuwen LA Geldhof V Pasut A Carmeliet P . COVID-19: the vasculature unleashed. Nat Rev Immunol. (2020) 20:389–91. doi: 10.1038/s41577-020-0343-0, PMID: 32439870 PMC7240244

[146] Fogarty H Townsend L Morrin H Ahmad A Comerford C Karampini E . Persistent endotheliopathy in the pathogenesis of long COVID syndrome. J Thromb Haemost. (2021) 19:2546–53. doi: 10.1111/jth.15490, PMID: 34375505 PMC8420256

[147] Wong AC Devason AS Umana IC Cox TO Dohnalová L Litichevskiy L . Serotonin reduction in post-acute sequelae of viral infection. Cell. (2023) 186:4851–4867.e20. doi: 10.1016/j.cell.2023.09.013, PMID: 37848036 PMC11227373

[148] Vijayakumar B Boustani K Ogger PP Papadaki A Tonkin J Orton CM . Immuno-proteomic profiling reveals aberrant immune cell regulation in the airways of individuals with ongoing post-COVID-19 respiratory disease. Immunity. (2022) 55:542–556.e5. doi: 10.1016/j.immuni.2022.01.017, PMID: 35151371 PMC8789571

[149] Patel MA Knauer MJ Nicholson M Daley M Van Nynatten LR Martin C . Elevated vascular transformation blood biomarkers in Long-COVID indicate angiogenesis as a key pathophysiological mechanism. Mol Med. (2022) 28:1–12. doi: 10.1186/s10020-022-00548-8, PMID: 36217108 PMC9549814

[150] Buonsenso D Sorrentino S Ferretti A Morello R Valentini P Di Gennaro L . Circulating activated platelets in children with long covid: A case-controlled preliminary observation. Pediatr Infect Dis J. (2024). doi: 10.1097/INF.0000000000004470, PMID: 39018475 PMC11542966

[151] Wang SSY Chee K Wong SW Tan GB Ang H Leung BP . Increased platelet activation demonstrated by elevated CD36 and P-selectin expression in 1-year post-recovered COVID-19 patients. Semin Thromb Hemost. (2023) 49:561–4. doi: 10.1055/s-0043-1762578, PMID: 36781153

[152] He F Huang B Bomsel M . Persistence of SARS-coV-2 in platelets and megakaryocytes in long COVID - CROI conference (2024). Available online at: https://www.croiconference.org/abstract/persistence-of-sars-cov-2-in-platelets-and-megakaryocytes-in-long-covid/ (Accessed January 3, 2025).

[153] Al-kuraishy HM Al-Gareeb AI Qusti S Alshammari EM Gyebi GA Batiha GES . Covid-19-induced dysautonomia: A menace of sympathetic storm. ASN Neuro. (2021) 13. doi: 10.1177/17590914211057635, PMID: 34755562 PMC8586167

[154] Spudich S Nath A . Nervous system consequences of COVID-19. Science. (2022) 375:267–9. doi: 10.1007/s00401-020-02166-2, PMID: 35050660

[155] Hugon J Queneau M Sanchez Ortiz M Msika EF Farid K Paquet C . Cognitive decline and brainstem hypometabolism in long COVID: A case series. (2022). doi: 10.1002/brb3.2513, PMID: 35290729 PMC9014998

[156] Song WJ Hui CKM Hull JH Birring SS McGarvey L Mazzone SB . Confronting COVID-19-associated cough and the post-COVID syndrome: role of viral neurotropism, neuroinflammation, and neuroimmune responses. Lancet Respir Med. (2021) 9:533–44. doi: 10.1016/S2213-2600(21)00125-9, PMID: 33857435 PMC8041436

[157] Castanares-Zapatero D Chalon P Kohn L Dauvrin M Detollenaere J Maertens de Noordhout C . Pathophysiology and mechanism of long COVID: a comprehensive review. Ann Med. (2022) 54:1473–87. doi: 10.1080/07853890.2022.2076901, PMID: 35594336 PMC9132392

[158] Zhang BZ Chu H Han S Shuai H Deng J Hu YF . SARS-CoV-2 infects human neural progenitor cells and brain organoids. Cell Res. (2020) 30:928–31. doi: 10.1038/s41422-020-0390-x, PMID: 32753756 PMC7399356

[159] Stein SR Ramelli SC Grazioli A Chung JY Singh M Yinda CK . SARS-CoV-2 infection and persistence in the human body and brain at autopsy. Nature. (2022) 612:758–63. doi: 10.1038/s41586-022-05542-y, PMID: 36517603 PMC9749650

[160] Peluso MJ Deeks SG Mustapic M Kapogiannis D Henrich TJ Lu S . SARS-coV-2 and mitochondrial proteins in neural-derived exosomes of COVID-19. Ann Neurol. (2022) 91:772–81. doi: 10.1002/ANA.26350, PMID: 35285072 PMC9082480

[161] Rutkai I Mayer MG Hellmers LM Ning B Huang Z Monjure CJ . Neuropathology and virus in brain of SARS-CoV-2 infected non-human primates. Nat Commun. (2022) 13:1–13. doi: 10.1038/s41467-022-29440-z, PMID: 35365631 PMC8975902

[162] Matschke J Lütgehetmann M Hagel C Sperhake JP Schröder AS Edler C . Neuropathology of patients with COVID-19 in Germany: a post-mortem case series. Lancet Neurol. (2020) 19:919–29. doi: 10.1016/S1474-4422(20)30308-2, PMID: 33031735 PMC7535629

[163] Visser D Golla SSV Verfaillie SCJ Coomans EM Rikken RM Van De Giessen EM . Long COVID is associated with extensive *in-vivo* neuroinflammation on [18F]DPA-714 PET. medRxiv. (2022). doi: 10.1101/2022.06.02.22275916, PMID: 41887800

[164] Bortolato B F. Carvalho A K. Soczynska J I. Perini G S. McIntyre R . The involvement of TNF-α in cognitive dysfunction associated with major depressive disorder: an opportunity for domain specific treatments. Curr Neuropharmacol. (2015) 13:558–76. doi: 10.2174/1570159X13666150630171433, PMID: 26467407 PMC4761629

[165] Reichard RR Kashani KB Boire NA Constantopoulos E Guo Y Lucchinetti CF . Neuropathology of COVID-19: a spectrum of vascular and acute disseminated encephalomyelitis (ADEM)-like pathology. Acta Neuropathol. (2020) 140:1–6. doi: 10.1007/s00401-020-02166-2, PMID: 32449057 PMC7245994

[166] Romero-Sánchez CM Díaz-Maroto I Fernández-Díaz E Sánchez-Larsen Á Layos-Romero A García-García J . Neurologic manifestations in hospitalized patients with COVID-19: The ALBACOVID registry. Neurology. (2020) 95:E1060–70. doi: 10.1212/WNL.0000000000009937, PMID: 32482845 PMC7668545

[167] Aubart M Roux CJ Durrleman C Gins C Hully M Kossorotoff M . Neuroinflammatory disease following severe acute respiratory syndrome coronavirus 2 infection in children. J Pediatr. (2022) 247:22–28.e2. doi: 10.1016/j.jpeds.2022.05.018, PMID: 35577119 PMC9106400

[168] Reiken S Sittenfeld L Dridi H Liu Y Liu X Marks AR . Alzheimer’s-like signaling in brains of COVID-19 patients. Alzheimer’s Dem. (2022) 18:955–65. doi: 10.1002/ALZ.12558, PMID: 35112786 PMC9011576

[169] Ameres M Brandstetter S Toncheva AA Kabesch M Leppert D Kuhle J . Association of neuronal injury blood marker neurofilament light chain with mild-to-moderate COVID-19. J Neurol. (2020) 267:3476. doi: 10.1007/S00415-020-10050-Y, PMID: 32647900 PMC7345451

[170] Kanberg N Ashton NJ Andersson LM Yilmaz A Lindh M Nilsson S . Neurochemical evidence of astrocytic and neuronal injury commonly found in COVID-19. Neurology. (2020) 95:E1754–9. doi: 10.1212/WNL.0000000000010111, PMID: 32546655

[171] Alvarez M Trent E Goncalves BDS Pereira DG Puri R Frazier NA . Cognitive dysfunction associated with COVID-19: Prognostic role of circulating biomarkers and microRNAs. Front Aging Neurosci. (2022) 14:1020092. doi: 10.3389/FNAGI.2022.1020092, PMID: 36268187 PMC9577202

[172] Sun B Tang N Peluso MJ Iyer NS Torres L Donatelli JL . Characterization and biomarker analyses of post-COVID-19 complications and neurological manifestations. Cells. (2021) 10:386. doi: 10.3390/CELLS10020386, PMID: 33668514 PMC7918597

[173] Charnley M Islam S Bindra GK Engwirda J Ratcliffe J Zhou J . Neurotoxic amyloidogenic peptides in the proteome of SARS-COV2: potential implications for neurological symptoms in COVID-19. Nat Commun. (2022) 13:1–11. doi: 10.1038/s41467-022-30932-1, PMID: 35697699 PMC9189797

[174] Villaume WA . Marginal BH4 deficiencies, iNOS, and self-perpetuating oxidative stress in post-acute sequelae of Covid-19. Med Hypotheses. (2022) 163:110842. doi: 10.1016/J.MEHY.2022.110842, PMID: 35431403 PMC9006446

[175] Guedj E Campion JY Dudouet P Kaphan E Bregeon F Tissot-Dupont H . 18F-FDG brain PET hypometabolism in patients with long COVID. Eur J Nucl Med Mol Imaging. (2021) 48:2823–33. doi: 10.1007/s00259-021-05215-4, PMID: 33501506 PMC7837643

[176] Cavaleri D Bartoli F Capogrosso CA Guzzi P Moretti F Riboldi I . Blood concentrations of neopterin and biopterin in subjects with depression: A systematic review and meta-analysis. Prog Neuropsychopharmacol Biol Psychiatry. (2023) 120:110633. doi: 10.1016/J.PNPBP.2022.110633, PMID: 36089162

[177] Appelman B Charlton BT Goulding RP Kerkhoff TJ Breedveld EA Noort W . Muscle abnormalities worsen after post-exertional malaise in long COVID. Nat Commun. (2024) 15:17. doi: 10.1038/s41467-023-44432-3, PMID: 38177128 PMC10766651

[178] Guarnieri JW Dybas JM Fazelinia H Kim MS Frere J Zhang Y . Core mitochondrial genes are down-regulated during SARS-CoV-2 infection of rodent and human hosts. Sci Trans Med. (2023) 15(708). Available online at: https://www.science.org. 10.1126/scitranslmed.abq1533 PMC11624572 37556555

[179] Paul BD Lemle MD Komaroff AL Snyder SH . Redox imbalance links COVID-19 and myalgic encephalomyelitis/chronic fatigue syndrome. Proc Natl Acad Sci Utd States America. (2021) 118:e2024358118. doi: 10.1073/pnas.2024358118, PMID: 34400495 PMC8403932

[180] Pozzi A . COVID-19 and mitochondrial non-coding RNAs: new insights from published data. Front Physiol. (2022) 12:805005/BIBTEX. doi: 10.3389/fphys.2021.805005, PMID: 35185603 PMC8856670

[181] Díaz-Resendiz KJG Benitez-Trinidad AB Covantes-Rosales CE Toledo-Ibarra GA Ortiz-Lazareno PC Girón-Pérez DA . Loss of mitochondrial membrane potential (ΔΨm) in leucocytes as post-COVID-19 sequelae. J Leukoc Biol. (2022) 112:23–9. doi: 10.1002/JLB.3MA0322-279RRR, PMID: 35355308 PMC9088601

[182] Guntur VP Nemkov T de Boer E Mohning MP Baraghoshi D Cendali FI . Signatures of mitochondrial dysfunction and impaired fatty acid metabolism in plasma of patients with post-acute sequelae of COVID-19 (PASC). Metabolites. (2022) 12. doi: 10.3390/metabo12111026, PMID: 36355108 PMC9699059

[183] Tyrkalska SD Pérez-Sanz F Franco-Martínez L Rubio CP Tvarijonaviciute A Martínez-Subiela S . Salivary biomarkers as pioneering indicators for diagnosis and severity stratification of pediatric long COVID. Front Cell Infect Microbiol. (2024) 14:1396263. doi: 10.3389/fcimb.2024.1396263, PMID: 38881733 PMC11176444

[184] Wood E Hall KH Tate W . Role of mitochondria, oxidative stress and the response to antioxidants in myalgic encephalomyelitis/chronic fatigue syndrome: A possible approach to SARS-CoV-2 “long-haulers”? Chronic diseases and translational medicine. (2021) 7:14–26. doi: 10.1016/J.CDTM.2020.11.002, PMID: 33251031 PMC7680046

[185] Di Girolamo FG Fiotti N Sisto UG Nunnari A Colla S Mearelli F . Skeletal muscle in hypoxia and inflammation: insights on the COVID-19 pandemic. Front Nutr. (2022) 9:865402/BIBTEX. doi: 10.3389/fnut.2022.865402, PMID: 35529457 PMC9072827

[186] Shin J Toyoda S Nishitani S Onodera T Fukuda S Kita S . SARS-CoV-2 infection impairs the insulin/IGF signaling pathway in the lung, liver, adipose tissue, and pancreatic cells via IRF1. Metab: Clin Exp. (2022) 133:155236. doi: 10.1016/j.metabol.2022.155236, PMID: 35688210 PMC9173833

[187] Tittel SR Rosenbauer J Kamrath C Ziegler J Reschke F Hammersen J . Did the COVID-19 lockdown affect the incidence of pediatric type 1 diabetes in Germany? Diabetes Care. (2020) 43:e172–3. doi: 10.2337/dc20-1633, PMID: 32826282 PMC7576433

[188] Trieu C Sunil B Ashraf AP Cooper J Yarbrough A Pinninti S . SARS-CoV-2 infection in hospitalized children with type 1 and type 2 diabetes. J Clin Trans Endocrinol. (2021) 26:100271. doi: 10.1016/J.JCTE.2021.100271, PMID: 34729361 PMC8553361

[189] Unsworth R Wallace S Oliver NS Yeung S Kshirsagar A Naidu H . New-onset type 1 diabetes in children during COVID-19: multicenter regional findings in the U.K. Diabetes Care. (2020) 43:e170–1. doi: 10.2337/dc20-1551, PMID: 32816997

[190] Suwanwongse K Shabarek N . Newly diagnosed diabetes mellitus, DKA, and COVID-19: Causality or coincidence? A report of three cases. J Med Virol. (2021) 93:1150–3. doi: 10.1002/JMV.26339, PMID: 32706395 PMC7404645

[191] Montefusco L Ben Nasr M D’Addio F Loretelli C Rossi A Pastore I . Acute and long-term disruption of glycometabolic control after SARS-CoV-2 infection. Nat Metab. (2021) 3:774–85. doi: 10.1038/s42255-021-00407-6, PMID: 34035524 PMC9931026

[192] Fernández-Ortega M Ponce-Rosas ER Muñiz-Salinas DA Rodríguez-Mendoza O Nájera Chávez P Sánchez-Pozos V . Cognitive dysfunction, diabetes mellitus 2 and arterial hypertension: Sequelae up to one year of COVID-19. Trav Med Infect Dis. (2023) 52:102553. doi: 10.1016/J.TMAID.2023.102553, PMID: 36805019 PMC9981335

[193] Gentile S Strollo F Mambro A Ceriello A . COVID-19, ketoacidosis and new-onset diabetes: Are there possible cause and effect relationships among them? Diab Obes Metab. (2020) 22:2507–8. doi: 10.1111/DOM.14170, PMID: 32790021 PMC7436911

[194] Giron LB Peluso MJ Ding J Kenny G Zilberstein NF Koshy J . Markers of fungal translocation are elevated during post-acute sequelae of SARS-CoV-2 and induce NF-κB signaling. JCI Insight. (2022) 7. doi: 10.1172/JCI.INSIGHT.164813, PMID: 35727635 PMC9462470

[195] Bizjak DA Stangl M Börner N Bösch F Durner J Drunin G . Kynurenine serves as useful biomarker in acute, Long- and Post-COVID-19 diagnostics. Front Immunol. (2022) 13:1004545. doi: 10.3389/FIMMU.2022.1004545, PMID: 36211365 PMC9537769

[196] Cysique LA Jakabek D Bracken SG Allen-Davidian Y Heng B Chow S . The kynurenine pathway relates to post-acute COVID-19 objective cognitive impairment and PASC. Ann Clin Trans Neurol. (2023) 10:1338–52. doi: 10.1002/ACN3.51825, PMID: 37318955 PMC10424655

[197] Seo SK Kwon B . Immune regulation through tryptophan metabolism. Exp Mol Med. (2023) 55:1371–9. doi: 10.1038/s12276-023-01028-7, PMID: 37394584 PMC10394086

[198] Blackett JW Sun Y Purpura L Margolis KG Elkind MSV O’Byrne S . Decreased gut microbiome tryptophan metabolism and serotonergic signaling in patients with persistent mental health and gastrointestinal symptoms after COVID-19. Clin Trans Gastroenterol. (2022) 13:e00524. doi: 10.14309/CTG.0000000000000524, PMID: 36049050 PMC9624499

[199] Rosser EC Piper CJM Matei DE Blair PA Rendeiro AF Orford M . Microbiota-derived metabolites suppress arthritis by amplifying aryl-hydrocarbon receptor activation in regulatory B cells. Cell Metab. (2020) 31:837–851.e10. doi: 10.1016/j.cmet.2020.03.003, PMID: 32213346 PMC7156916

[200] Turski WA Wnorowski A Turski GN Turski CA Turski L . AhR and IDO1 in pathogenesis of Covid-19 and the “Systemic AhR Activation Syndrome:” a translational review and therapeutic perspectives. Restor Neurol Neurosci. (2020) 38:343–54. doi: 10.3233/RNN-201042, PMID: 32597823 PMC7592680

[201] Sarangi P . Role of indoleamine 2, 3-dioxygenase 1 in immunosuppression of breast cancer. Cancer Pathog Ther. (2023). doi: 10.1016/j.cpt.2023.11.001, PMID: 39371092 PMC11447360

[202] Kaiser H Parker E Hamrick MW . Kynurenine signaling through the aryl hydrocarbon receptor: Implications for aging and healthspan. Exp Gerontol. (2020) 130:110797. doi: 10.1016/J.EXGER.2019.110797, PMID: 31786316 PMC7899131

[203] Malina HZ Hess OM . Xanthurenic acid translocates proapoptotic Bcl-2 family proteins into mitochondria and impairs mitochondrial function. BMC Cell Biol. (2004) 5:14. doi: 10.1186/1471-2121-5-14, PMID: 15068490 PMC400728

[204] Hoffmann M Kleine-Weber H Schroeder S Krüger N Herrler T Erichsen S . SARS-coV-2 cell entry depends on ACE2 and TMPRSS2 and is blocked by a clinically proven protease inhibitor. Cell. (2020) 181:271–280.e8. doi: 10.1016/j.cell.2020.02.052, PMID: 32142651 PMC7102627

[205] Singer D Camargo SMR Ramadan T Schäfer M Mariotta L Herzog B . Defective intestinal amino acid absorption in Ace2 null mice. Am J Physiol - Gastrointest Liv Physiol. (2012) 303:686–95. doi: 10.1152/ajpgi.00140.2012, PMID: 22790597

[206] Berger M Gray JA Roth BL . The expanded biology of serotonin. Annu Rev Med. (2009) 60:355–66. doi: 10.1146/annurev.med.60.042307.110802, PMID: 19630576 PMC5864293

[207] Kupari J Häring M Agirre E Castelo-Branco G Ernfors P . An atlas of vagal sensory neurons and their molecular specialization. Cell Rep. (2019) 27:2508–2523.e4. doi: 10.1016/j.celrep.2019.04.096, PMID: 31116992 PMC6533201

[208] Dotan A Mahroum N Bogdanos DP Shoenfeld Y . COVID-19 as an infectome paradigm of autoimmunity. J Allergy Clin Immunol. (2022) 149:63–4. doi: 10.1016/j.jaci.2021.11.009, PMID: 34826507 PMC8610602

[209] Liu Q Mak JWY Su Q Yeoh YK Lui GCY Ng SSS . Gut microbiota dynamics in a prospective cohort of patients with post-acute COVID-19 syndrome. Gut. (2022) 71:544–52. doi: 10.1136/gutjnl-2021-325989, PMID: 35082169

[210] Venzon M Cadwell K . COVID-19 and the forgotten organ: prolonged changes to the metabolic output of the gut microbiome. Gastroenterology. (2022) 162:394–6. doi: 10.1053/j.gastro.2021.11.017, PMID: 34800482 PMC8596650

[211] Zhang F Lau RI Liu Q Su Q Chan FKL Ng SC . Gut microbiota in COVID-19: key microbial changes, potential mechanisms and clinical applications. Nat Rev Gastroenterol Hepatol. (2022) 20:323–37. doi: 10.1038/s41575-022-00698-4, PMID: 36271144 PMC9589856

[212] Zuo T Liu Q Zhang F Lui GCY Tso EYK Yeoh YK . Depicting SARS-CoV-2 faecal viral activity in association with gut microbiota composition in patients with COVID-19. Gut. (2021) 70:276–84. doi: 10.1136/gutjnl-2020-322294, PMID: 32690600 PMC7385744

[213] Brooks EF Bhatt AS . The gut microbiome: A missing link in understanding the gastrointestinal manifestations of COVID-19? Cold Spring Harbor Mol Case Stud. (2021) 7. doi: 10.1101/MCS.A006031, PMID: 33593727 PMC8040733

[214] Van Doorn AS Meijer B Frampton CMA Barclay ML De Boer NKH . Systematic review with meta-analysis: SARS-CoV-2 stool testing and the potential for faecal-oral transmission. Aliment Pharmacol Ther. (2020) 52:1276–88. doi: 10.1111/apt.16036, PMID: 32852082 PMC7461227

[215] Padua-Zamora MdAP Rey MdKLR Tan-Lim MMCSC Gregorio MM PhD GEV . Gastrointestinal and hepatic manifestations of COVID-19 in children: A systematic review and meta-analysis. Acta Med Phil. (2024) 58. doi: 10.47895/amp.v58i7.7054, PMID: 38882920 PMC11168955

[216] Donati Zeppa S Agostini D Piccoli G Stocchi V Sestili P . Gut microbiota status in COVID-19: an unrecognized player? Front Cell Infect Microbiol. (2020) 10:576551/BIBTEX. doi: 10.3389/fcimb.2020.576551, PMID: 33324572 PMC7725702

[217] Zuo T Zhang F Lui GCY Yeoh YK Li AYL Zhan H . Alterations in gut microbiota of patients with COVID-19 during time of hospitalization. Gastroenterology. (2020) 159:944–955.e8. doi: 10.1053/j.gastro.2020.05.048, PMID: 32442562 PMC7237927

[218] Penninger JM Grant MB Sung JJY . The role of angiotensin converting enzyme 2 in modulating gut microbiota, intestinal inflammation, and coronavirus infection. Gastroenterology. (2021) 160:39–46. doi: 10.1053/j.gastro.2020.07.067, PMID: 33130103 PMC7836226

[219] Wang H Wang H Sun Y Ren Z Zhu W Li A . Potential associations between microbiome and COVID-19. Front Med. (2021) 8:785496. doi: 10.3389/fmed.2021.785496, PMID: 35004750 PMC8727742

[220] Vestad B Ueland T Lerum TV Dahl TB Holm K Barratt-Due A . Respiratory dysfunction three months after severe COVID-19 is associated with gut microbiota alterations. J Internal Med. (2022) 291:801–12. doi: 10.1111/JOIM.13458, PMID: 35212063 PMC9115297

[221] Yeoh YK Zuo T Lui GCY Zhang F Liu Q Li AYL . Gut microbiota composition reflects disease severity and dysfunctional immune responses in patients with COVID-19. Gut. (2021) 70:698–706. doi: 10.1136/gutjnl-2020-323020, PMID: 33431578 PMC7804842

[222] Scott NA Pearmain L Knight SB Brand O Morgan DJ Jagger C . Monocyte migration profiles define disease severity in acute COVID-19 and unique features of long COVID. Eur Respir J. (2023) 61. doi: 10.1183/13993003.02226-2022, PMID: 36922030 PMC10040898

[223] Mendes De Almeida V Engel DF Ricci MF Cruz S Santos Lopes Í Alves DA . Gut microbiota from patients with COVID-19 cause alterations in mice that resemble post-COVID symptoms. (2023). doi: 10.1080/19490976.2023.2249146, PMID: 37668317 PMC10481883

[224] Carneiro VL Littlefield KM Watson R Palmer BE Lozupone C . Inflammation-associated gut microbiome in postacute sequelae of SARS-CoV-2 points towards new therapeutic targets. Gut. (2023) 73:376–8. doi: 10.1136/gutjnl-2022-328757, PMID: 36717218 PMC10850647

[225] Wais T Hasan M Rai V Agrawal DK . Gut-brain communication in COVID-19: molecular mechanisms, mediators, biomarkers, and therapeutics. Expert Rev Clin Immunol. (2022) 18:947–60. doi: 10.1080/1744666X.2022.2105697, PMID: 35868344 PMC9388545

[226] Riedel CU Foata F Philippe D Adolfsson O Eikmanns BJ Blum S . Anti-inflammatory effects of bifidobacteria by inhibition of LPS-induced NF-&kappa;B activation. World J Gastroenterol. (2006) 12:3729–35. doi: 10.3748/wjg.v12.i23.3729, PMID: 16773690 PMC4087466

[227] Duranti S Ruiz L Lugli GA Tames H Milani C Mancabelli L . Bifidobacterium adolescentis as a key member of the human gut microbiota in the production of GABA. Sci Rep. (2020) 10:14112. doi: 10.1038/s41598-020-70986-z, PMID: 32839473 PMC7445748

[228] Dang AT Marsland BJ . Microbes, metabolites, and the gut–lung axis. Mucosal Immunol. (2019) 12:843–50. doi: 10.1038/s41385-019-0160-6, PMID: 30976087

[229] Glass MC Glass DR Oliveria JP Mbiribindi B Esquivel CO Krams SM . Human IL-10-producing B cells have diverse states that are induced from multiple B cell subsets. Cell Rep. (2022) 39. doi: 10.1016/j.celrep.2022.110728, PMID: 35443184 PMC9107325

[230] Arpaia N Campbell C Fan X Dikiy S Van Der Veeken J Deroos P . Metabolites produced by commensal bacteria promote peripheral regulatory T-cell generation. Nature. (2013) 504:451–5. doi: 10.1038/nature12726, PMID: 24226773 PMC3869884

[231] Alameddine J Godefroy E Papargyris L Sarrabayrouse G Tabiasco J Bridonneau C . Faecalibacterium prausnitzii skews human DC to prime IL10-producing T cells through TLR2/6/JNK signaling and IL-10, IL-27, CD39, and IDO-1 induction. Front Immunol. (2019) 10:143. doi: 10.3389/fimmu.2019.00143, PMID: 30787928 PMC6373781

[232] Silva YP Bernardi A Frozza RL . The role of short-chain fatty acids from gut microbiota in gut-brain communication. Front Endocrinol. (2020) 11:25. doi: 10.3389/fendo.2020.00025, PMID: 32082260 PMC7005631

[233] Bradley KC Finsterbusch K Schnepf D Crotta S Llorian M Davidson S . Microbiota-driven tonic interferon signals in lung stromal cells protect from influenza virus infection. Cell Rep. (2019) 28:245–256.e4. doi: 10.1016/J.CELREP.2019.05.105, PMID: 31269444

[234] Deriu E Boxx GM He X Pan C Benavidez SD Cen L . Influenza virus affects intestinal microbiota and secondary salmonella infection in the gut through type I interferons. PloS Pathog. (2016) 12:1–26. doi: 10.1371/journal.ppat.1005572, PMID: 27149619 PMC4858270

[235] Noij L Hashimoto S Winkel A Lap C Brackel-Kosterink C Teela L . Persistent COVID-19 symptoms and quality of life among the Dutch pediatric population. Eur Respir Soc. (2022) 8:165. doi: 10.1183/23120541.LSC-2022.165, PMID: 41918948

[236] Camporesi A Vetrugno L Buonsenso D . COVID-19 and children: reflections after three years. Children. (2023) 11:10. doi: 10.3390/children11010010, PMID: 38275431 PMC10814962

[237] Munblit D Nicholson T Akrami A Apfelbacher C Chen J De Groote W . A core outcome set for post-COVID-19 condition in adults for use in clinical practice and research: an international Delphi consensus study. Lancet Respir Med. (2022) 10:715–24. doi: 10.1016/S2213-2600(22)00169-2, PMID: 35714658 PMC9197249

[238] Greenhalgh T Sivan M Perlowski A Nikolich JŽ . Long COVID: a clinical update. Lancet. (2024) 404:707–24. doi: 10.1016/S0140-6736(24)01136-X, PMID: 39096925

[239] Subramanian D Vittala A Chen X Julien C Acosta S Rusin C . Stratification of pediatric COVID-19 cases using inflammatory biomarker profiling and machine learning. J Clin Med. (2023) 12. doi: 10.3390/jcm12175435, PMID: 37685502 PMC10487951

[240] Gross RS Thaweethai T Kleinman LC Snowden JN Rosenzweig EB Milner JD . Characterizing long COVID in children and adolescents. JAMA. (2024) 332:1174–88. doi: 10.1001/jama.2024.12747, PMID: 39196964 PMC11339705

[241] Reese JT Blau H Casiraghi E Bergquist T Loomba JJ Callahan TJ . Generalisable long COVID subtypes: Findings from the NIH N3C and RECOVER programmes. eBioMedicine. (2023) 87:1–17. doi: 10.1016/j.ebiom.2022.104413, PMID: 36563487 PMC9769411

[242] Kenny G McCann K O’Brien C Savinelli S Tinago W Yousif O . Identification of distinct long COVID clinical phenotypes through cluster analysis of self-reported symptoms. Open Forum Infect Dis. (2022) 9:ofac060. doi: 10.1093/ofid/ofac060, PMID: 35265728 PMC8900926

[243] Mateu L Tebe C Loste C Santos JR Lladós G López C . Determinants of the onset and prognosis of the post-COVID-19 condition: a 2-year prospective observational cohort study. Lancet Reg Health - Eur. (2023) 33:100724. doi: 10.1016/j.lanepe.2023.100724, PMID: 37954002 PMC10636281

[244] Noij LCE Blankestijn JM Lap CR Van Houten MA Biesbroek G Der ZAHMV . Clinical-based phenotypes in children with pediatric post-COVID-19 condition. World J Pediatr. (2024) 20:682–91. doi: 10.1007/s12519-024-00805-2, PMID: 38664324 PMC11269322

[245] Blair PW Brandsma J Chenoweth J Richard SA Epsi NJ Mehta R . Distinct blood inflammatory biomarker clusters stratify host phenotypes during the middle phase of COVID-19. Sci Rep. (2022) 12:22471. doi: 10.1038/s41598-022-26965-7, PMID: 36577783 PMC9795438

[246] Qi E Courcoubetis G Liljegren E Herrera E Nguyen N Nadri M . Investigation of liquid biopsy analytes in peripheral blood of individuals after SARS-CoV-2 infection. eBioMedicine. (2023) 90. doi: 10.1016/j.ebiom.2023.104519, PMID: 36921564 PMC10008671

[247] Captur G Moon JC Topriceanu CC Joy G Swadling L Hallqvist J . Plasma proteomic signature predicts who will get persistent symptoms following SARS-CoV-2 infection. eBioMedicine. (2022) 85. doi: 10.1016/j.ebiom.2022.104293, PMID: 36182629 PMC9515404

[248] Wang K Khoramjoo M Srinivasan K Gordon PMK Mandal R Jackson D . Sequential multi-omics analysis identifies clinical phenotypes and predictive biomarkers for long COVID. Cell Rep Med. (2023) 4. doi: 10.1016/j.xcrm.2023.101254, PMID: 37890487 PMC10694626

[249] An AY Baghela A Zhang PGY Blimkie TM Gauthier J Kaufmann DE . Post-COVID symptoms are associated with endotypes reflecting poor inflammatory and hemostatic modulation. Front Immunol. (2023) 14:1243689. doi: 10.3389/fimmu.2023.1243689, PMID: 37680625 PMC10482103

